# Assessment of myocardial viscoelasticity with Brillouin spectroscopy in myocardial infarction and aortic stenosis models

**DOI:** 10.1038/s41598-021-00661-4

**Published:** 2021-11-01

**Authors:** María Villalba-Orero, Rafael J. Jiménez-Riobóo, Nuria Gontán, Daniel Sanderson, Marina López-Olañeta, Pablo García-Pavía, Manuel Desco, Enrique Lara-Pezzi, Maria Victoria Gómez-Gaviro

**Affiliations:** 1 Centro Nacional de Investigaciones Cardiovasculares (CNIC), Melchor Fernández Almagro, 3, 28029 Madrid, Spain; 2 Instituto de Ciencia de Materiales de Madrid, Consejo Superior de Investigaciones Científicas (CSIC), Madrid, Spain; 3 Instituto de Investigación Sanitaria Gregorio Marañón, Madrid, Spain; 4 Hospital Puerta de Hierro Majadahonda, Madrid, Spain; 5 Centro de Investigación Biomédica en Red Cardiovascular (CIBERCV), Madrid, Spain; 6 Universidad Francisco de Vitoria (UFV), Pozuelo de Alarcon, Spain; 7 Centro de Investigación Biomédica en Red Salud Mental (CIBERSAM), Madrid, Spain; 8 Departamento de Bioingeniería e Ingeniería Aeroespacial, Universidad Carlos III, Madrid, Spain; 9 Hospital General Universitario Gregorio Marañón, Doctor Esquerdo 46, 28007 Madrid, Spain

**Keywords:** Biological techniques, Biophysics, Cardiology

## Abstract

Heart diseases are associated with changes in the biomechanical properties of the myocardial wall. However, there is no modality available to assess myocardial stiffness directly. Brillouin microspectroscopy (mBS) is a consolidated mechanical characterization technique, applied to the study of the viscoelastic and elastic behavior of biological samples and may be a valuable tool for assessing the viscoelastic properties of the cardiac tissue. In this work, viscosity and elasticity were assessed using mBS in heart samples obtained from healthy and unhealthy mice (n = 6 per group). Speckle-tracking echocardiography (STE) was performed to evaluate heart deformation. We found that mBS was able to detect changes in stiffness in the ventricles in healthy myocardium. The right ventricle showed reduced stiffness, in agreement with its increased compliance. mBS measurements correlated strongly with STE data, highlighting the association between displacement and stiffness in myocardial regions. This correlation was lost in pathological conditions studied. The scar region in the infarcted heart presented changes in stiffness when compared to the rest of the heart, and the hypertrophied left ventricle showed increased stiffness following aortic stenosis, compared to the right ventricle. We demonstrate that mBS can be applied to determine myocardial stiffness, that measurements correlate with functional parameters and that they change with disease.

## Introduction

Cardiac function relies not only on the ability of the sarcomere to contract and relax, but also on the preservation of the shape and structure of the organ^1^. The mechanical properties of the myocardial tissue determine how sarcomeres shorten and develop force independently of the preexisting loading conditions^2,3^. Heart diseases are often associated with dysregulation of these mechanical properties, which lead to remodeling of the ventricular wall, tissue stiffness and progressively to dysfunction^2,4^. Specifically, changes in myocardial viscoelasticity are linked to dynamic stiffness and heart disease^5^. However, assessing these properties directly and at a local scale is challenging.

Myocardial infarction is caused by the blockage of a coronary artery, which deprives cardiac cells from the necessary oxygen and nutrients. This results in massive cardiomyocyte death and a steep decline in cardiac contraction^6^. Due to the limited ability of the myocardium to regenerate, dead cardiomyocytes are substituted by a fibrotic tissue, mainly composed of collagens. While this fibrotic tissue prevents cardiac rupture, it cannot contract and its mechanical properties differ considerably from those of the healthy myocardium. Changes in the stiffness of the heart progressively lead to myocardial remodeling, further cardiac dysfunction and, eventually, heart failure^7^.

Similarly, myocardial hypertrophy is initially developed as a compensatory response to aortic stenosis or to other pathological conditions demanding increased contractile capacity^8^. However, the sustained thickening of the ventricular walls and the production of perivascular and interstitial fibrosis progressively lead to myocardial remodeling, and to relaxation and contraction defects^9^.

Both pathological processes are associated with changes in the biomechanical properties of the cardiac muscle caused both by overstretching of the sarcomere and by excessive production of extracellular matrix. Mechanical properties of the myocardium have been assessed before by techniques that either do not provide the same microscopic resolution, such as echocardiography or cardiac magnetic resonance imaging, or require contact, such as atomic force microscopy^10^. While these techniques provide information about heart regions or the organ as a whole, they have limited spatial resolution (up to 1.2 to 3 mm)^11^. The development of myocardial fibrosis in response to pathological stimuli is often heterogeneous. Therefore, accurately determining the biomechanical properties of different myocardial regions is necessary to have a more complete understanding of the complex behavior of the tissue in disease conditions. Furthermore, determining the relationship between viscoelasticity and the tissue composition would provide valuable information for disease screening and follow-up^12^. Thus, methods to understand the heterogeneity of the mechanical properties of the tissue at a micrometer scale resolution and their contribution to the organ’s function and structure in a contactless manner are desirable.

Brillouin microspectroscopy (mBS) is a consolidated mechanical characterization technique in the field of condensed matter in solid state physics^13,14^. In the life sciences, it has recently been applied to the study of the viscoelastic and elastic behavior of biological samples in the micro/GHz scale. Based on the principle of Brillouin light scattering (BLS), mBS determines the elastic moduli of biological materials by stimulating the sample with visible light and measuring the thermally induced acoustic waves or phonons that propagate throughout the sample^15^. Although the first applications of mBS in biology appeared in the late seventies^16,17^, it was not until recently that the field started to flourish, following the first report of a VIPA (virtually imaged phased array) spectrometer^18^. Compared to other technologies used to assess biomechanical properties, mBS has the advantage of being a contactless method that does not destroy or change the tissue. In addition, it is label-free and can be combined with other imaging techniques^19,20^ to provide a full picture of the structural and mechanical properties of the tissue.

Reports of Brillouin applications in biology include characterization of β-amyloid plaques^21,22^ and atherosclerotic plaque^23^ in mouse models, melanoma diagnosis^24,25^, and a wide range of experiments in the ophthalmological field, in which the technique shows great promise. Clinically viable devices for in vivo assessment of cornea stiffness have already been tested in humans^26,27^. In vivo monitoring of cranial tube development and the spinal growth and repair in live zebrafish have also been reported^28,29^.

We have recently showed that mBS can be used to detect potential changes in myocardial tissue associated with tissue clearing methods^30^. However, the changes in elasticity or viscosity in the context of heart disease and their association with functional parameters have not been shown. Here we provide the first application of mBS to the measurement of these properties in cardiac homeostasis and disease. We show that myocardial elasticity measured by mBS is lower in the right ventricle. We also show that mBS frequency shifts correlate with speckle tracking values determined by echocardiography. Interestingly, this association is lost in heart disease. We believe that the mBS provides valuable information that, in a label-free manner, improves the understanding of biomechanical changes that impact cardiac structure and function, and their dysregulation in disease.

## Results

### Application of Brillouin microspectroscopy to the study of adult mouse myocardium

**Figure 1:**
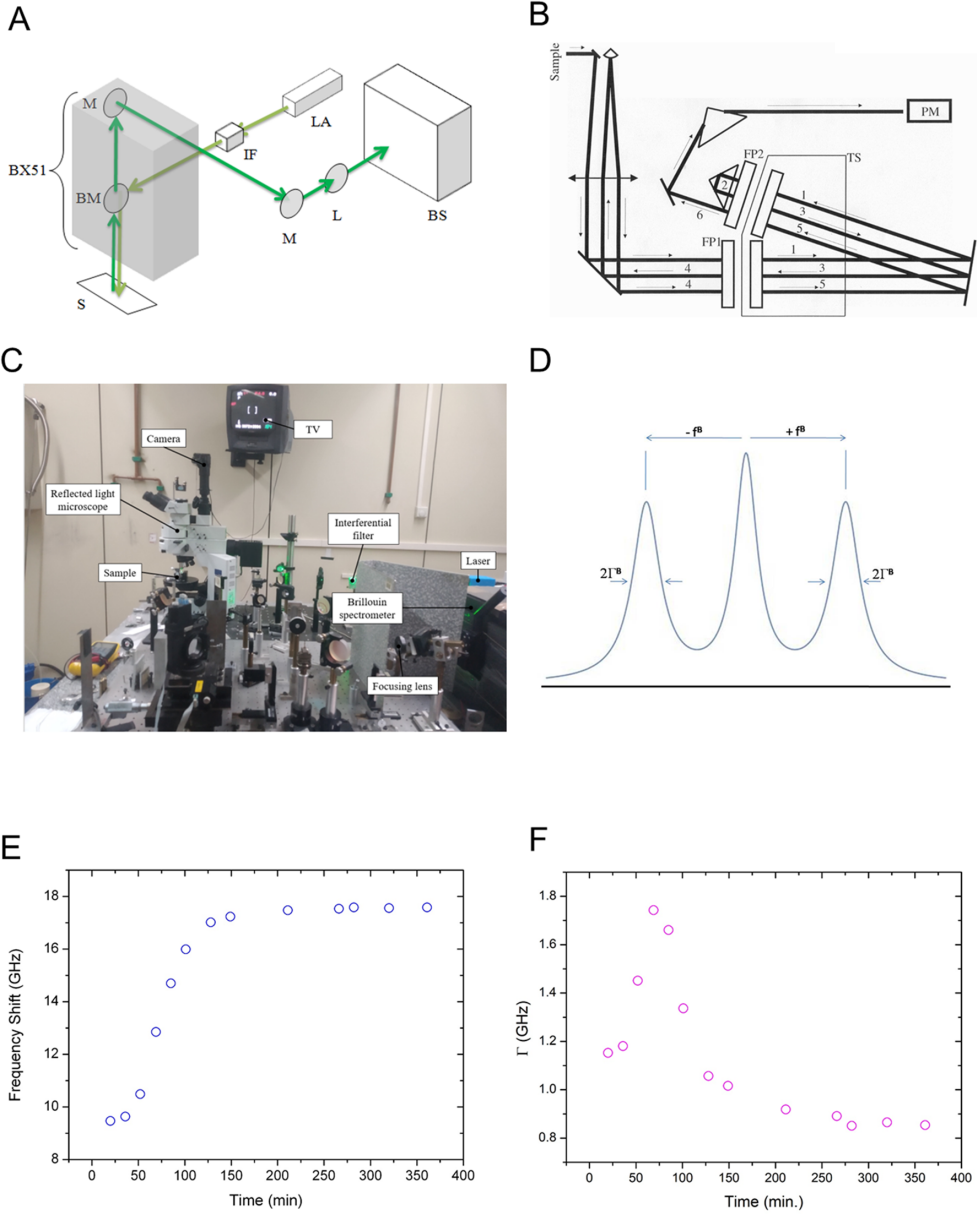
Schematics of the Brillouin spectroscopy setup and experimental arrangement. (**A**–**C**) Experimental set up used to perform Brillouin microspectroscopy (mBS) measurements combined a home modified Olympus BX51 reflected light microscope with a 3 + 3 Tandem Fabry–Pérot spectrometer. (**A**) Light path schematics of the mBS experiment from laser light source to Brillouin spectrometer passing through the Olympus BX51 microscope. (**B**) Principle of the 3 + 3 Tandem Fabry–Pérot based Brillouin spectrometer. (**C**) Actual picture of the experimental setup. (**D**) Ideal mBS spectrum for a liquid. The Brillouin frequency shift f^180^ is defined as f^180^ = ω^B^/2π. (**E**, **F**) mBS frequency shift (f^180^, **E**) and peak width (HWHM, **F**) were measured during sample drying for different time lengths.

We have previously shown that mBS can be applied to cleared myocardial samples^30^. However, its use with non-cleared cardiac tissue has not been demonstrated. In order to confirm the feasibility of the Brillouin spectroscopy to measure viscoelasticity of myocardial sections, a protocol for the preparation of mouse heart samples was developed, taking into account the appropriate thickness of the sample so that the sections were sufficiently transparent for light to transverse the tissue completely. Optimal section thickness to avoid myocardium fibers falling apart when drying was found to be 500 µm. Using this thickness also facilitated the obtention of whole unbroken samples, reducing the chances of right ventricle or infarcted tissue rupture during vibratome slicing. Establishing clear time periods for sample drying made the measuring process more efficient. As shown in Fig. 1E, F, samples needed to be air-dried for a minimum of 280 min to reach a plateau in mBS f180 and HWHM measurements.

**Figure 2:**
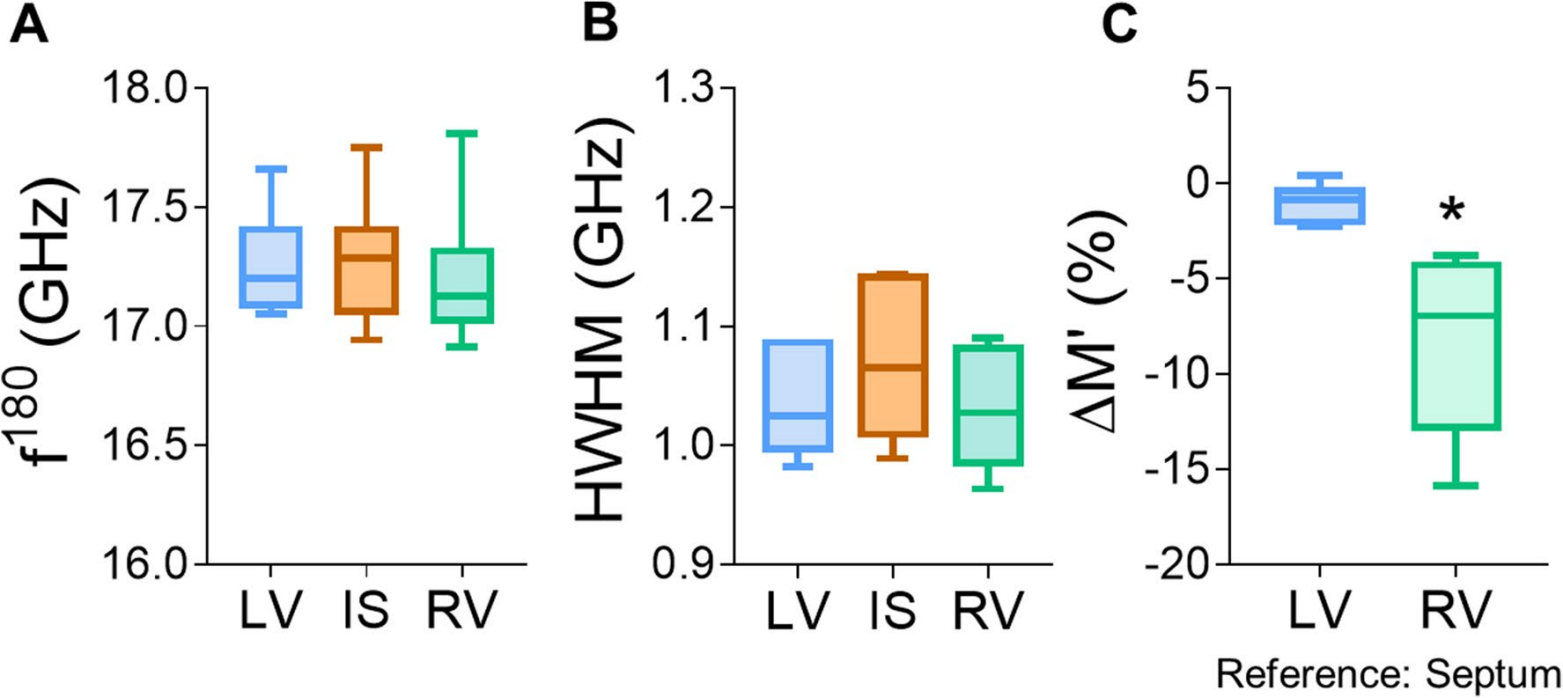
Brillouin microspectroscopy can be applied to the study of the adult myocardium. (**A**, **B**) Frequency shift (f^180^, **A**) and peak width (HWHM, **B**) were measured in different cardiac segments in uninjured heart sections using mBS. (**C**) Average of the relative variation of the elastic modulus M′ in the LV and the RV relative to the IS in each sample. Statistical analysis was performed using 1-way ANOVA and Tukey post-hoc tests (n = 6 independent points in each segment). *LV* left ventricle, *IS* interventricular septum, *RV* right ventricle.

To assess viscoelastic properties of adult, uninjured, mouse hearts, we measured mBS scattering frequency shift (f^180^) and peak linewidth (HWHM) in multiple points of the left and right ventricle (LV and RV, respectively) as well as in the interventricular septum (IS; Fig. S11-6 and Tables S1–S6). As shown in Fig. 2, no significant differences in the absolute value of f^180^ (LV, 17.26 GHz; IS, 17.28 GHz, and RV, 17.20 GHz; Fig. 2A) or HWHM were observed when considering cardiac regions as a whole (LV: 1.03 GHz; IS: 1.07 GHz and RV: 1.03 GHz, Fig. 2B).

Although the exact longitudinal elastic modulus (M′) cannot be calculated without knowing the density and the refractive index of the sample, and the scattering angle of the incident light, the Lorentz–Lorentz relation for non-strongly absorbing materials states that the refractive index n^2^ approximately scales with the mass density so that variations in these parameters will cancel each other out^14,31^, thereby allowing the calculation of the relative variation of M’ in different regions within the same sample. As shown in Fig. 2C, the RV free wall showed a significantly lower elastic module M’ than the LV, on average, using the IS as a reference in each sample. This result suggests reduced stiffness in this ventricle, in agreement with its known higher compliance.

### Correlation between Brillouin frequency shifts and speckle tracking echocardiography in uninjured hearts

**Figure 3:**
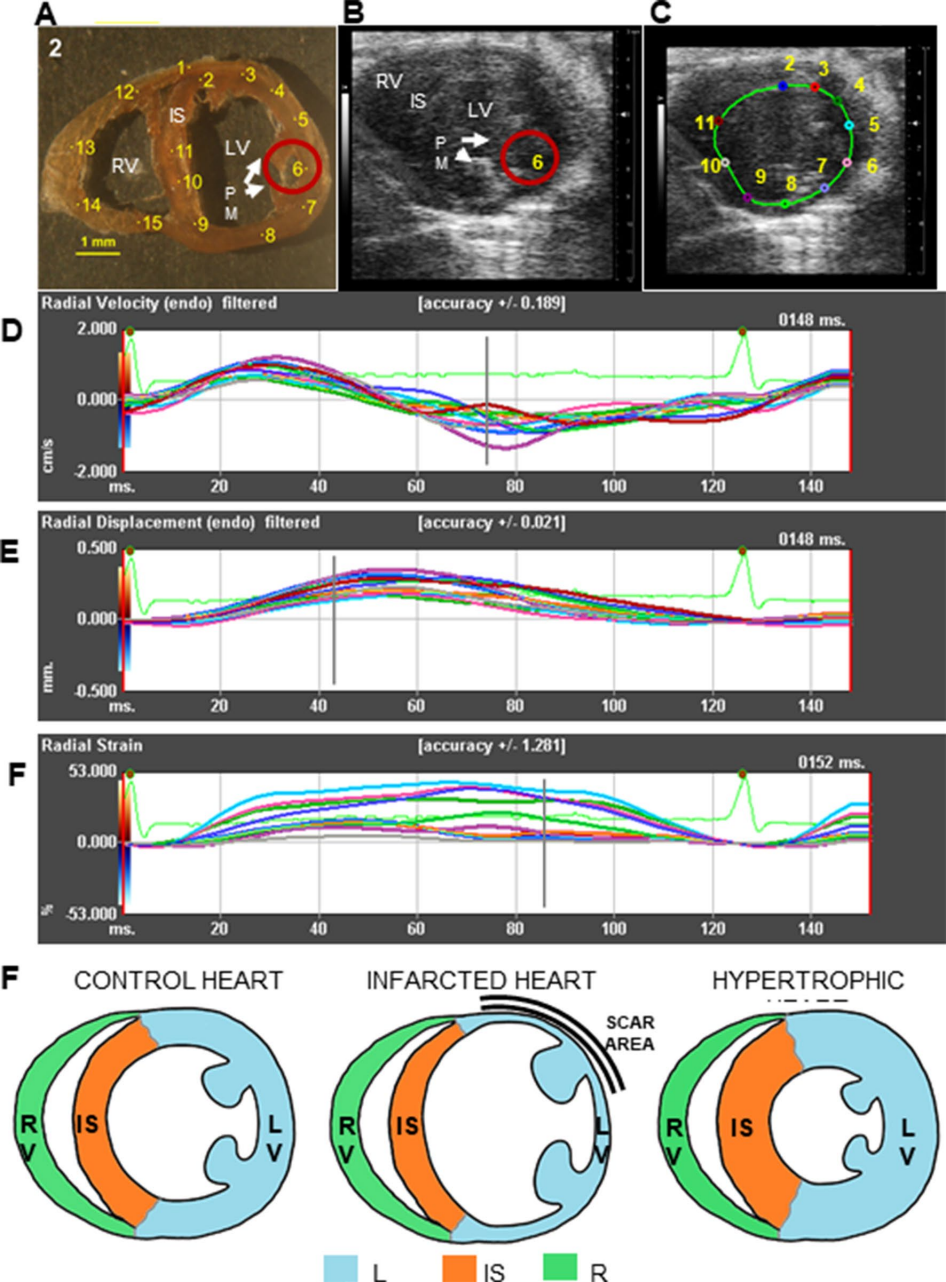
Speckle tracking echocardiography and corresponding segments. (**A**) Myocardial points analysed by mBS in a representative control heart. (**B**, **C**) Short axis echocardiography image (**B**) with the selected points to be tracked (**C**). (**D–F**) Representative radial velocity (**D**), radial displacement (**E**) and radial strain (**F**) measured along time for each selected point. (**G**) Heart segments analysed in the study. *RV* right ventricle, *IS* interventricular septum, *LV* left ventricle, *ms* milliseconds.

**Figure 4:**
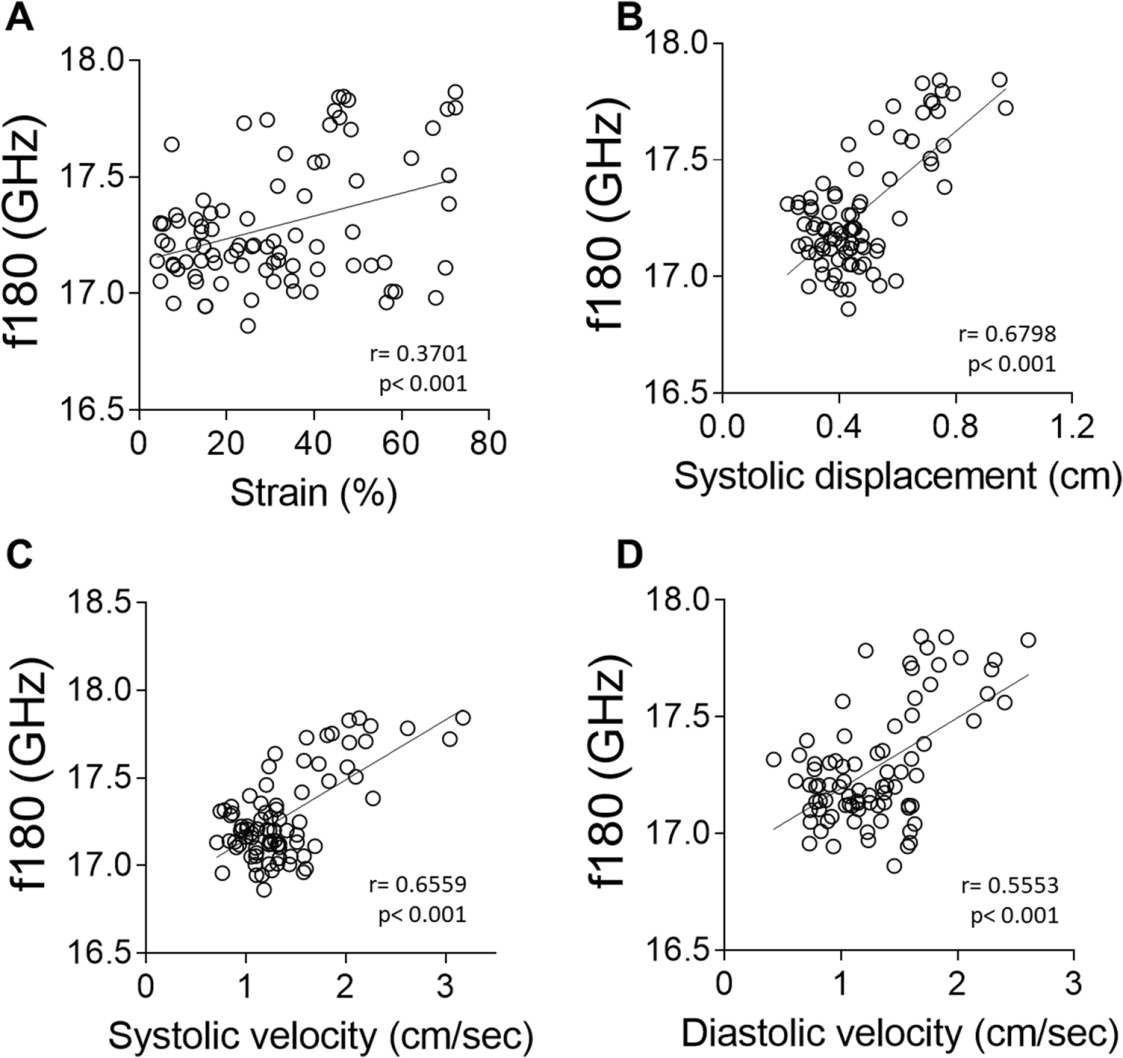
Brillouin frequency shifts correlate with radial wall deformation and motion indexes measured by echocardiography. (**A**–**D**) Correlation between mBS frequency shift and radial strain (**A**), radial systolic displacement (**B**), radial systolic velocity (**C**), and radial diastolic velocity (**D**) measured by 2D speckle tracking with echocardiography. Statistical analysis was performed using Pearson correlation coefficient (r).

We next investigated the correlation between elasticity and viscosity assessed by mBS and ventricular wall motion and deformation during cardiac movement assessed by 2D speckle tracking (ST) echocardiography (Fig. 3A–G). Speckle tracking works by tracking the movement of natural acoustic echoic points or “speckles” which are present in ultrasound tissue images, to measure wall strain or deformation when force is applied, offering a measure of myocardial contractility. It indirectly estimates the elastic features of the different cardiac segments by calculating the segmental cardiac velocity, displacement and deformation indices (strain). Figure 4 shows correlations between mBS deformation indices and strain, displacement, diastolic, and systolic velocity using ST echocardiography. Although there is no significant correlation between strain and f^180^ (Fig. 4A), we found that f^180^ correlated with tissue displacement and velocity (Fig. 4B–D), suggesting that variations in tissue elasticity measured by mBS in the different regions are associated with myocardial strain and motility.

### Brillouin frequency shifts in scar tissue following myocardial infarction

**Figure 5:**
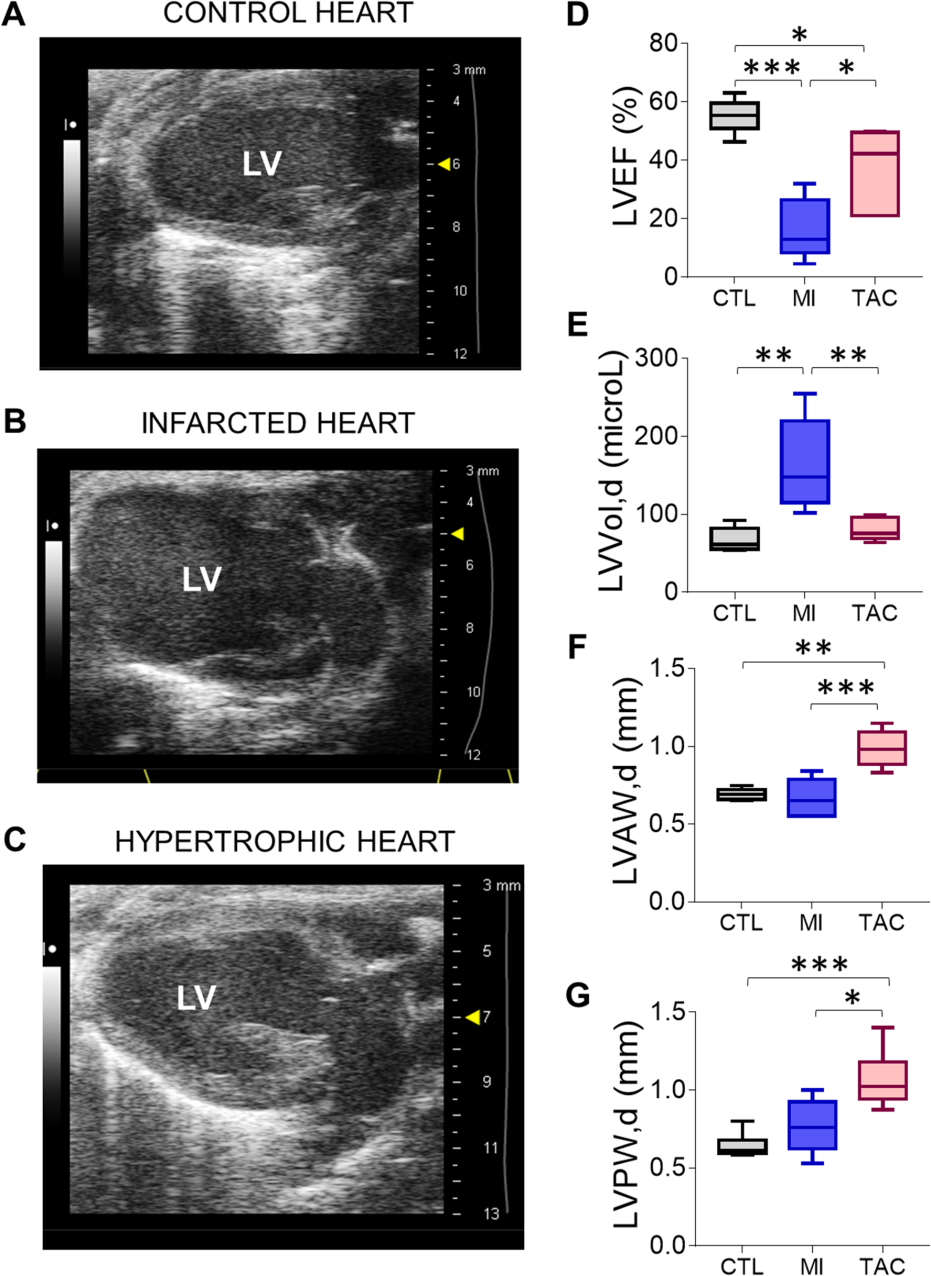
Assessment of cardiac function by echocardiography. (**A**–**C**) Representative echocardiography images of a control heart (**A**), an infarcted heart (**B**), and a hypertrophic heart (**C**). (**D**–**G**) Functional and structural parameters were quantified by echocardiography, including left ventricular ejection fraction (LVEF; **D**), left ventricular end diastolic volume (LVVol,d; **E**), left ventricular anterior wall thickness in diastole (LVAW,d; **F**), and left ventricular posterior wall thickness in diastole (LVPW,d; **G**). *p < 0.05, **p < 0.01, ***p < 0.001; one-way ANOVA followed by Bonferroni post-test. n = 6 per group.

**Figure 6:**
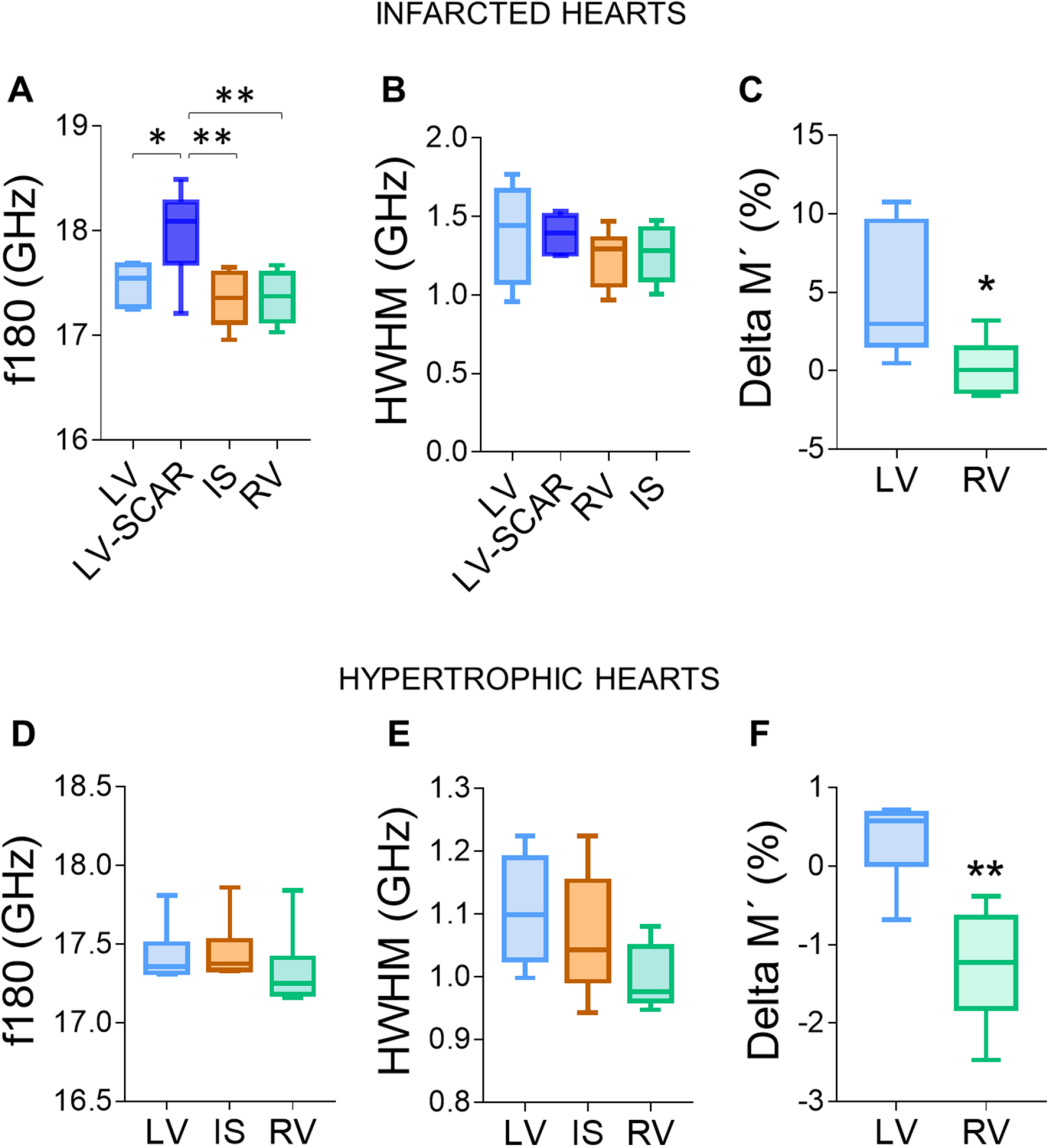
Brillouin frequency shift is increased in the scar region of infarcted hearts. (**A**–**C**) Myocardial infarction was induced by permanent ligation of the left descending coronary artery and mice were sacrificed 28 days later. Brillouin frequency shift (**A**), peak width (**B**) and the average of the relative variation of the elastic modulus M′ were measured in different cardiac segments. (**D–F**), Cardiac hypertrophy was induced by transaortic banding and f180, HWHM and the relative variation of M′ were measured as in (**A**–**C**). *p < 0.05; **p < 0.01. 1-way ANOVA and Tukey post-hoc test (**A**, **B**, **D**, **E**) or t-test (**C**, **F**). n = 6 in each segment. *LV* left ventricle without scar, *LV-scar* infarct scar in the left ventricle, *IS* interventricular septum, *RV* right ventricle.

We next investigated whether mBS could detect changes in the viscoelasticity of the myocardial tissue associated with myocardial infarction. The assessment was performed 28 days post-injury, when cardiac remodeling was already evident. We induced myocardial infarction by permanent coronary artery occlusion, which, as expected^32^, caused left ventricular overload and systolic dysfunction (Fig. 5A–E). Pathological hypertrophy was induced by TAC, and was characterized by thickening of the ventricular wall and systolic dysfunction (Fig. 5C–G)^33^. Twenty eight days after left coronary artery ligation, the mBS frequency spectra of different points in the heart sections were acquired, including infarcted (LV-scar) and non-infarcted (LV) LV regions, IS and RV (Fig. S2, Supplementary Tables S7–S12). The infarcted area showed a significantly higher f^180^ compared to the other cardiac regions (Fig. 6A), whereas no significant differences in HWHM were detected between the different regions (Fig. 6B). Hearts subjected to ischemia showed a higher f^180^ and HWHM than control, uninjured hearts, but these only reached statistical significance for the LV, which in this case included infarcted and non-infarcted regions (Fig. S3A,B). As in uninjured hearts, the average of the relative difference of the M’ modulus in the RV compared to the IS was significantly lower than that of the LV (Fig. 6C). Taken together, these results suggest that, in general, the infarcted wall of the left ventricle is stiffer and therefore presents less deformation, which would be associated with a replacement of the dead cardiomyocytes by a fibrotic scar.

### mBS detects a shift in myocardial elasticity as a result of transaortic constriction

Transthoracic aortic constriction was used to induce LV pressure overload, which progressed and led to LV hypertrophy accompanied by diffuse fibrosis. No significant differences between the different cardiac regions were found for f^180^ and HWHM in absolute terms (Fig. 6D,E; Supplementary Tables S13–S18). However, the RV showed a significant decrease in its elastic modulus minus relative to that of the IS, compared to that of the LV (Fig. 6F). Hearts subjected to LV pressure overload presented an increased f^180^ compared to control hearts, although differences did not reach significance, possibly due to the limited sample size (Fig. S3C). No significant differences between hypertrophic and uninjured hearts were found in HWHM (Figs. S3D, S4).

### Correlation between Brillouin frequency shifts and speckle tracking echocardiography is lost in diseased hearts

**Figure 7:**
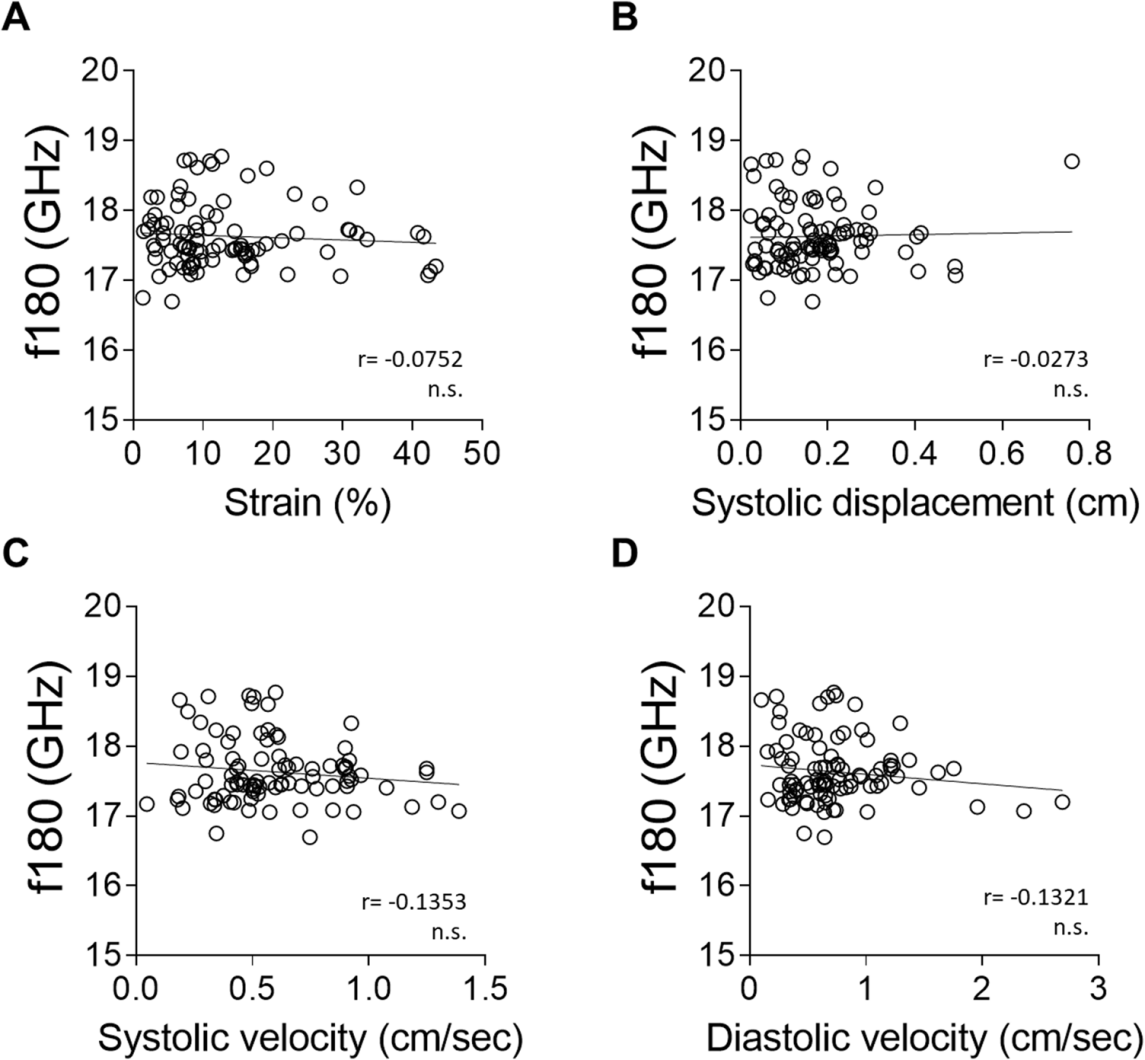
The correlation between Brillouin frequency shifts and radial wall deformation and motion indexes measured by echocardiography is lost after myocardial infarction. (**A**–**D**) Correlation between mBS frequency shift and radial strain (**A**), radial systolic displacement (**B**), radial systolic velocity (**C**), and radial diastolic velocity (**D**) measured by 2D speckle tracking with echocardiography. Statistical analysis was performed using Pearson correlation coefficient (r).

**Figure 8:**
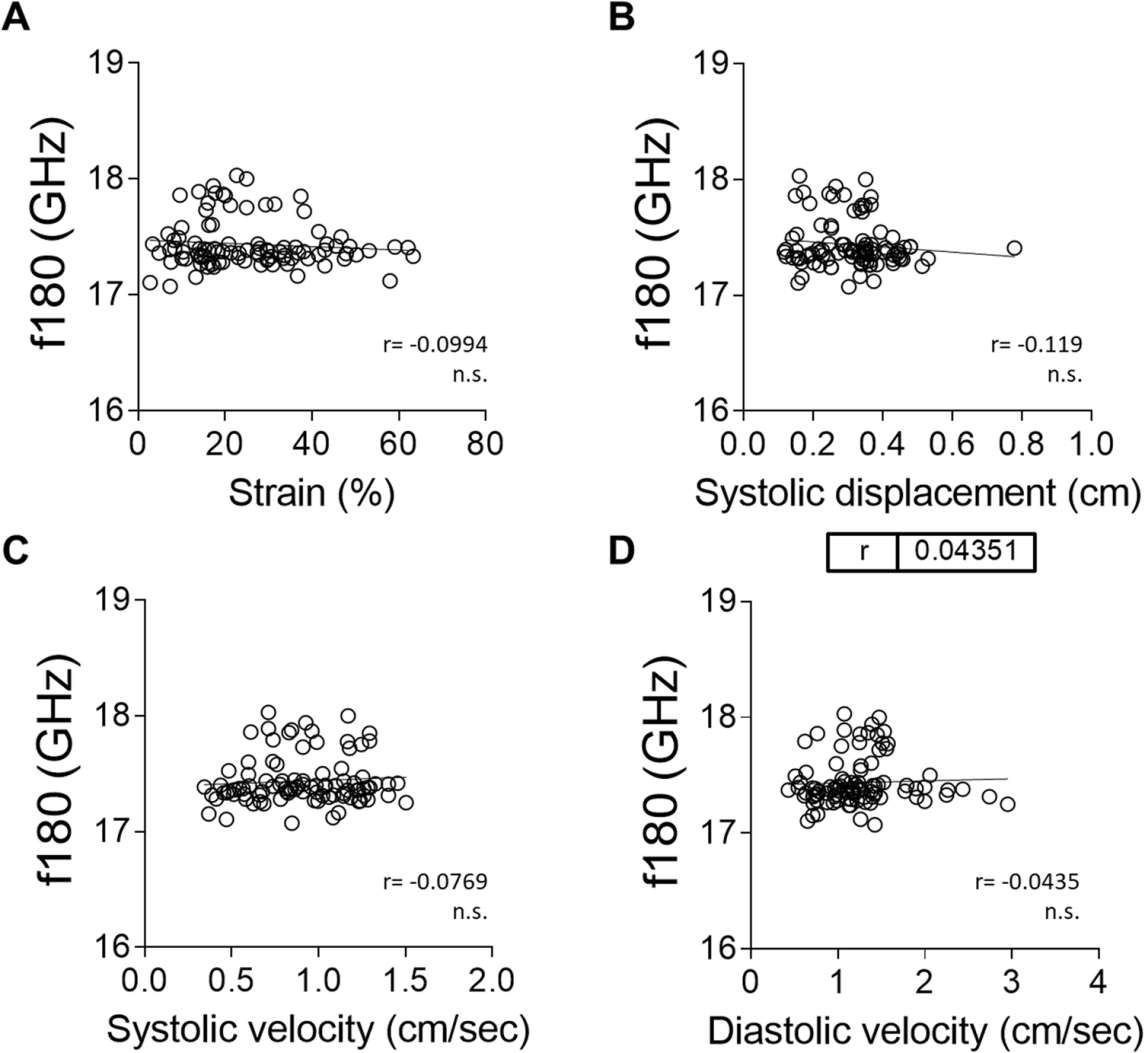
There is no significant correlation between Brillouin frequency shifts and radial wall deformation and motion indexes measured by echocardiography in the hypertrophic heart. (**A**–**D**) Correlation between mBS frequency shift and radial strain (**A**), radial systolic displacement (**B**), radial systolic velocity (**C**), and radial diastolic velocity (**D**) measured by 2D speckle tracking with echocardiography. Statistical analysis was performed using Pearson correlation coefficient (r).

We next investigated whether the correlation between elasticity measured by mBS frequency shift and myocardial motion and deformation measured by ST echocardiography were reproduced under pathological conditions. We found no correlation of f^180^ with strain rate, myocardial displacement or displacement velocity, neither in infarcted (Fig. 7A–D) nor in hypertrophic hearts (Fig. 8A–D), suggesting that the correlation between elasticity, motion and deformation is a hallmark of a healthy heart.

## Discussion

Left ventricular stiffening has been associated with cardiac dysfunction and heart failure^9^. Many cardiac pathologies can alter the global and regional dynamics in the heart, as the ventricular movements depend on the mechanical properties of the myocardial tissue. Left ventricular stiffening interferes with the local mechanical properties of the cellular microenvironment, leading to reduced compliance that will eventually affect the lungs, causing pulmonary hypertension, and right ventricle dysfunction^34^. Therefore, the development of analytical tools that enable the assessment of the viscoelastic properties of the heart is of the utmost importance.

In this work, we have evaluated the potential of mBS for the study of cardiac biomechanical properties in mouse models of myocardial infarction and pressure overload-induced hypertrophy. We developed a protocol for sample preparation, acquisition of mBS spectra to obtain frequency shift and linewidth from the Brillouin peaks of each probed area, and data analysis. We successfully mapped Brillouin frequency shifts in MI, TAC, and control samples using mBS. These measurements represent the first report of the Brillouin frequency shift of the diseased myocardium.

We found that the heart has heterogeneous elasticity. The free wall of the RV showed a significantly lower elastic module M’ than that of the LV, using the IS as a reference. This reduced stiffness agrees with the higher compliance in this ventricular wall and may have a strong impact on ventricular hemodynamics^35,36^. Since mBS measurements are uniaxial in the direction perpendicular to the sample and do not depend on the wall thickness, it is likely that the reduced stiffness is related to the particular sarcomere length-pressure curve relationship in the RV, rather than to a thinner ventricular wall.

We found a very good correlation between the Brillouin frequency shift and myocardial movement (systolic displacement, systolic and diastolic displacement velocities) in control hearts. We also found a significant correlation between Brillouin frequency shift and myocardial strain, indicating an association between elasticity and deformation during cardiac contraction. Together, these results indicate that changes in elasticity between different ventricular regions, as measured by mBS, are associated with functional differences, further highlighting the relevance of this biomechanical feature in cardiac contraction.

Interestingly, we did not see this correlation in either of the two models of heart disease, myocardial infarction and aortic stenosis. Velocity and displacement generally describe wall motion whereas strain characterizes wall deformation. Thus, a moving object that changes its position over time shows displacement but does not necessarily show deformation (strain) if all its parts move with the same velocity^37^. Lack of uniformity in cardiac movement velocity may impact strain measured by echocardiography. Although changes in elasticity, as measured by mBS, will also have an impact on contraction and relaxation, their relative impact may not correlate. In any case, it is tempting to speculate that the lack of correlation between Brillouin frequency shift and myocardial strain and/or displacement may be in itself a hallmark disease, due to changes in elasticity not being paralleled by the ability of the myocardium to be deformed.

We showed that mBS is able to detect variations in the elastic behavior of the myocardium that are developed as a result of pathology. Particular success was achieved in distinguishing between infarcted and healthy myocardium. mBS showed a significant increase in Brillouin frequency shift in the scar region compared to control hearts and to the remote (uninjured) myocardium. This increase is in agreement with other reports showing an increased Brillouin frequency shift with increased collagen concentration, both in tissues and biopolymers^23,38^, and highlights the ability of mBS to detect changes in elasticity associated with fibrotic processes.

We found that the left ventricle of hypertrophic hearts has increased Brillouin frequency shifts compared to control left ventricles and compared to the right ventricle. These changes would be associated with the hypertrophy of individual cardiomyocytes in the LV as a result of aortic stenosis^8^, and with the resulting increased stiffness of the left ventricle^8,9^. In addition, changes in elasticity may also be associated with the interstitial collagen deposition in this disease model^7^.

We also detected differences in viscosity, evidenced by the variations observed in the Brillouin peak width between the different samples. In particular, we observed a significant increase in the peak width in infarcted hearts. The peak width is dependent on viscosity, density, and thermal conductivity, among others, and thus, changes with the kind of material composition. Myocardial infarction usually induces a replacement fibrosis after cell damage or necrosis the scar, but also leads to a general cardiac overload, which causes an activation of inflammatory and pro-fibrotic pathways that globally affect the heart and results in proliferation of different cell lines. The changes in peak width may reflect the different tissue composition in infarcted hearts. Unfortunately, HWHM is much more affected by external conditions than f^180^. These external conditions can include among others the numerical aperture of the microscope objective, better or worse focusing of the incident beam on the sample, the optical quality of the surface of the sample (smooth or rough), and its degree of transparency. All these external effects modify the magnitude of the width of the Brillouin peak and makes it difficult to obtain precise conclusions about the actual physical processes behind HWHM changes.

mBS measurements provide information about the biomechanical properties of the heart at a higher resolution than traditional techniques. Combined with its label-free and contactless nature, it represents a unique tool for high resolution, noninvasive mechanical assessment, as opposed to other available techniques which either do not reach microscopic resolution, as is the case of magnetic resonance, or require the application of physical forces to the sample, as it happens with atomic force microscopy (AFM), microrheology or magnetic tweezers. Elastography, an emerging tool to asses mechanical properties of biological tissues, requires parallel spatial resolution, like other acoustic imaging modalities, which limits accurate measurements^12^. It measures the compression and extension of the tissue in response to a force, which represents the tissue strain. Moreover, elastography has been proved to be successfully used in vivo as well as in ex vivo scenarios. Since the applied force is challenging to quantify, strain elastography cannot be considered a direct measure of tissue stiffness, as the absolute value of elasticity and Young´s Modulus cannot be determined. In addition, it depends on the operator´s pressure, presenting high intra and inter-operator variability^39^. Therefore, strain analysis of the myocardium using ultrasound is generally performed with speckle tracking techniques^40^, as we did in this report to validate mBS. The correlation between speckle tracking and mBS stiffness estimation argues in favour of the reliability of this technique. Another advantage of mBS is the capability to obtain information in parallel and perpendicular direction to the fibres that constitute the tissue, according to the sample preparation. Also, tissues don’t suffer plastic deformation as is the case with elastrography and the resolution of the mBS is very few micrometres.

The main limitation encountered during the experimental process was the long acquisition times, which ranged from minutes to hours depending on the optical quality of the surface for a single spectrum. Since measuring each sample took three to four days, the samples had to be stored at 4 °C in sealed microscopic slides to avoid possible damage. It would have been preferable for the laser light to impinge directly upon the myocardium rather than go through the microscopic slide glass coverslip, which might distort the signal slightly. Nevertheless, the glass coverslip was placed above all samples so any distortion that might have occurred would have been common to all measurements and should not have affected this study, in which information was extracted from direct comparison between data points. Importantly, although ST echocardiography is currently the most widespread technique to quantify myocardial deformation^41^ and correlates with tissue stiffness, the intrinsic myocardial strain does not exactly reflect the tissue stiffness. mBS applications have been described for mechanobiology and disease screening in many organs and pathologies. This tool is gaining importance in cancer research, and the eyes, joints, uterus, vessels have been addressed to detect structural changes at the molecular level^12^. The use of mBS in the heart will complement traditional approaches to provide a bigger picture of the biomechanical, structural and functional properties of the myocardium. It holds the potential to provide new insight into the underlying mechanisms of cardiac pathology and will hopefully prove useful in improving our understanding of myocardial dysfunction, becoming a powerful tool for the assessment and development of new therapeutic approaches. This is particularly important in certain pathological cardiac conditions, like HFpEF or restrictive cardiomyopathy, dominated by impaired relaxation and increased stiffness and for which there are no therapies available.

## Material and methods

### Mice

All the experimental procedures have been performed in accordance with relevant named guidelines and regulations and all protocols were approved by the CNIC’s Institutional Animal Care and Research Advisory Committee of the Ethics Committee of the Regional Government of Madrid (PROEX177/17). Authors complied with ARRIVE guidelines.

A total of 18 male C57BL/6 mice, 8–10-weeks-old, weighting 25–30 g, housed in an air conditioned room with a 12 h light/dark cycle and free access to water and chow were used in this study. Mice were separated in 3 different groups, control group (CTL, n = 6), myocardial infarction group (MI, n = 6) and cardiac transaortic constriction group (TAC, n = 6).

### Surgical procedures

In both cardiac injury models, mice were anesthetized with sevoflurane (5% for induction, 2–3% for maintenance), and intubated using a 24-gauge intravenous catheter with a blunt end. Mice were mechanically ventilated using rodent ventilator (minivent 845) providing 160 breathing/min and a tidal volume of 250 μl. Mice were placed on a heating pad to maintain body temperature above 37 °C. The thorax and the skin incisions were closed with 6/0 silk sutures (Lorca Marín) and buprenorphine (0.01 mg/kg intraperitoneal, Buprex, Merck & Co. Inc) was administered for pain relief^32^. After the surgical procedure, mice were placed in a warm chamber until full consciousness was regained.

### MI model by left anterior descending coronary artery ligation (LAD-ligation)

A left thoracotomy was performed between the third and fourth ribs. After pericardiectomy, the proximal left descending artery (LAD) was permanently ligated using a 7/0 silk suture to induce myocardial infarction. The incision wound in the thorax was sutured, sevofluorane administration was stopped, and O_2_ was administered until reflexes were detected.

### Hypertrophy model by transverse aortic constriction (TAC)

The aortic arch was exposed by entering the pleural space above the first rib through a midline thoracotomy. The transverse aorta was isolated between the right and left carotid arteries and a 7–0 nylon suture ligature was tied around a 27-gaugle needle and the aorta to induce constriction. The needle was removed after ligation and the incision was sutured using a 7/0 silk. The sevoflurane vaporizer was closed and, once the animal regained reflex, oxygen was removed.

### Echocardiography measures

Transthoracic echocardiography was performed by an expert operator using a high-frequency ultrasound system (Vevo 2100, Visualsonics Inc, Canada) with a 40-MHz linear probe. After removing the hair of the thorax region, mice were lightly anesthetized with 0.5–2.0% isoflurane in 100% oxygen, adjusting the isoflurane delivery to maintain the heart rate at 450 ± 50 bpm. Mice were placed in supine position using a heating platform and warmed ultrasound gel was used to maintain normothermia. A base apex electrocardiogram (ECG) was used for continuous monitoring. Echocardiography was performed 28 days following LAD ligation or TAC surgery.

The left ventricle was assessed using standard bidimensional (2D) parasternal standard long and short axis views (LAX and SAX, respectively) and apical 4-chamber view as previously described^42^. Using the SAX, multiple planes were acquired from the ventricular base to the apex and stored. Images were then transferred to a computer and were blindly analyzed off-line using the Vevo 2100 workstation software, which performs speckle-tracking echocardiography (STE) analysis of the acquired 2D gray-scale echocardiographic loops.

Speckle tracking analyses were performed using the Vevo 2100 STE software by automatically delineating the endocardial and epicardial myocardial borders tracked frame-by-frame during systole and diastole. Manual adjustments were made as required in order to optimize border tracking. From these borders and their motion, radial systolic and diastolic velocity, displacement and strain were quantified selecting a point that was as close as possible to those selected for the Brillouin analysis. To easily identify the selected points for mBS evaluation, papillary muscles were used as a reference and the individual velocity, displacement and strain were quantified.

Velocity is a vectorial parameter with a direction and amplitude. Displacement (X_t_) is the time integral of the corresponding velocity:

$${\text{X}}_{{\text{t}}} = \int \limits_{ED}^{t} V\left( {t^{\prime }} \right)dt^{\prime }$$

Strain describes tissue deformation of an object normalized to its original shape and size. Calculations were performed by comparison of the speckles from frame to frame using a single reference length (L_0_) against subsequent deformation (Lagrangian strain):

$${\text{Strain }} = \frac{Lt - L0 }{{L0}}$$

being Lt the length at a given point in time and being the reference length (L_0_) taken at the end of the diastole.

### Perfusion, sacrifice and dissection

Animals were sacrificed by gradually filling the chamber with carbon dioxide. The heart was perfused intracardially with 15 ml of phosphate buffered saline (PBS) 1 × to flush blood out of the animal’s vessels, followed by 30 ml of 4% paraformaldehyde (PFA). The hearts were excised and incubated in 4% PFA 4% for 36 h 4 °C. Samples were then rinsed with PBS and stored in PBS at 4 °C until processed for Brillouin spectroscopy.

### Sample processing

Brillouin spectroscopy is highly sensitive to water content. Since mapping with the spectrometer took several days per sample, measurements were performed on dried samples to avoid having to account for water content variability. To this end, we developed the following protocol for sample processing to ensure that samples were completely dry. The tip of the heart apex was carefully sliced using a disposable laboratory blade to allow the entry of agarose into the ventricular lumen, which facilitates slicing with the vibratome without the sample breaking. Samples were then embedded in agarose (0.2 g agarose/10 ml distilled water) and sliced into 500 μm sections using a vibratome (Campdem Instruments). Slices with an intact right ventricle and appropriate representation of the infarct scar (for the myocardial infraction model) were chosen and mounted onto microscopic slides. A cover slip was placed on top of the sample without sealing. This facilitated transport and protected the samples from damage during the several days it takes to measure them. Transparent nail polish was applied on the sides to fix the cover slip. Samples were dried out at room temperature for 5 h and stored at 4 °C overnight.

### Brillouin spectroscopy setup

The experimental set up used to perform Brillouin microspectroscopy (mBS) measurements combined a home modified Olympus BX51 reflected light microscope with a 3 + 3 Tandem Fabry-Pérot spectrometer (J. Sandercock, Table Stable Ltd.). The light source was a DPSS laser with a wavelength of 532 nm and a light power below 1 mW on the sample. The microscope objective used to focus the incident light beam and to collect the scattered light simultaneously was an Olympus MPlan 10X with numerical aperture of 0.25. Schematics of the setup and the actual experimental arrangement can be seen in Fig. 1A–C.

Due to the microscope configuration, the scattering geometry used in the mBS experiments was the backscattering one. In this case the acoustic wave vector (q) is related to the laser wavelength (λ_0_)^43^:

$${\text{q}} = \frac{{4\uppi {\text{n}}}}{{\uplambda _{0} }}$$

being n the refractive index of the sample under investigation. An ideal BS spectrum for a liquid is shown in Fig. 1D. The mBS experiment delivered the Brillouin frequency shift f180 (f180 = ω^B^/2π), which combined with the acoustic wave vector, enabled us to compute the sound propagation velocity (v) in the physical medium studied:

$${\text{v = }}\frac{{\upomega ^{{\text{B}}} }}{{\text{q}}} = {\text{ }}\frac{{{\text{f}}^{{180}} \uplambda _{0} {\text{ }}}}{{{\text{2 n}}}}$$

The corresponding longitudinal elastic constant was obtained based on the mass density (ρ):

$${\text{M}}^{\prime } = \uprho {\text{v}}^{2} = \uprho \frac{{{\text{(f}}^{{180}} {\text{)}}^{2} \uplambda _{0}^{2} }}{{4{\text{n}}^{2} }}$$

This longitudinal elastic constant can be also found as the real part of the storage modulus M′. The determination of the elastic constant depends on the density and on the refractive index squared. In cases where the Lorenz–Lorentz relation holds, the effects of density and refractive index cancel and can be assumed that the elastic constant is proportional to the square of the Brillouin frequency shift^44,45^. However, this may not be true in complex biological systems, as is the case of tissues. Therefore we also used a magnitude independent of ρ and n, like the relative change in elastic constant that is directly related to the relative change in Brillouin frequency shift. This value enabled us to determine relative variations in the elastic modulus within each sample, and the average of these values within each group.

$$\frac{{\Delta {\text{ M}}^{\prime } }}{{{\text{M}}^{\prime } }}{\text{ 100 }} = {\text{2 }}\frac{{\Delta {\text{f}}^{{180}} }}{{{\text{f}}^{{180}} }}{\text{ 100}}$$

The Brillouin peak, was used as the value half width at half maximum (HWHM) was also measured by mBS. In simple homogeneous systems HWHM can be related to the longitudinal kinematic viscosity η_L_^46^.

$$\text{HWHM= }\frac{1}{{2}}{ \uprho }{\upeta}_{\text{L}}{{\text{q}}}^{2}$$

and thus to the imaginary part of the storage modulus M″ (M″ = ω^B^ η_L_).

### Data acquisition

To allow for direct comparison between spectra in order to elucidate the variations detected by mBS in the tissue, conditions were monitored and kept the same during all acquisitions. Printed photographs of each sample were labeled before each acquisition once the laser light had been focused onto an area of the sample to keep track of the location of the measured points. To identify the infarcted area (LV-SCAR) for labelling, we relied on pallor and thinness of ventricular wall when compared to the rest of the myocardium. The spectral acquisition was performed with the GHOST 7.0 software, (http://www.jrs-si.ch/downloads/ghost6.06.zip) and the laboratory temperature was monitored with a Pt1000 resistance thermometer. Due to the temperature-dependence of phonon velocity, temperature in the laboratory was kept constant at 25 °C ± 0.2 °C during acquisition at all times to minimize its influence. The mirror distance of the Fabry-Pérot interferometers was kept at 0.005 m, and the scanning voltage values were close to 0.05 V. Values were carefully monitored and recorded up to the fourth decimal for proper data fitting using a previously calculated scaling factor dependent on the FSR, the number of channels (512) and voltage controlling the parallel mirrors of the Fabry-Pérot. Heart samples were located under the microscope on a xy moving platform to change the point of incidence of light. A camera (Olympus C70) was attached to the microscope and connected to a TV to visualize the amplified image with white light in order to choose the point of focus for the laser beam. All three available Olympus objectives (X50, X20, X10, X5) were used for inspection of the sample; the X10 lens (numerical aperture (NA) = 0.25) was the one used for acquisition. The X10 objective focuses light onto areas of around 8 µm^2^ in samples with optically good surfaces, so it is to be expected that our areas of focus rounded the 10–15 µm^2^ range due to non-ideality. We took note of the location of each measurement in a printed photograph of the sample for region-based analysis. Spectral acquisition times ranged from 30 to 90 min depending on the optical quality offered by the surface of the chosen area. Since several days were needed to cover each sample, the microscopic slides were kept at 4 ºC when they were not being used. The samples were given at least 15 min out of the fridge at the beginning of each day to reach room temperature before starting to acquire any spectra in order to avoid temperature distortion.

### Data processing

f180, its error and HWHM and were extracted using the PeakFit 4.11 program (PeakFit 4.11: https://systatsoftware.com/) using the Deconvolution option for curve fitting, where the spectrum was deconvoluted with a Lorentzian response function followed by a Fourier filtering algorithm^46^. This process results in a spectrum of sharpened peaks that was fitted by least squares to a Lorentzian function plus a background function to account for the instrument’s response. We chose an exponential background in most cases, switching to a power or quadratic background when the algorithm failed to converge. Values for estimated parameters were converted to frequency using a scaling factor that related them through applied voltage.

OriginLab was used for calculation of the frequency shift f180, its error and HWHM. Assuming that Brillouin peaks are theoretically symmetric with respect to the Rayleigh central line, the overall parameter values for the spectrum were calculated following equations:

$${\text{f}}^{{180}} = \frac{{{\text{S}} - {\text{AS}}}}{2}\left( {{\text{channels}}} \right) \cdot {\text{ FE}}\left( {\frac{{{\text{GHz}}}}{{{\text{channel}} \cdot {\text{V}}}}} \right) \cdot {\text{ramp}}\left( {\text{V}} \right)$$

$${\text{HWHM}} = \frac{{{\text{S}} + {\text{AS}}}}{2}\left( {{\text{channels}}} \right) \cdot {\text{FE}}\left( {\frac{{{\text{GHz}}}}{{{\text{channel}} \cdot {\text{V}}}}} \right) \cdot {\text{ramp}}\left( {\text{V}} \right)$$

With S being the Brillouin shift for the Stokes peak, AS the Brillouin shift for the Anti-Stokes peak, ramp being the voltage supplied to the piezoelectric crystals of the Fabry-Pérot interferometer at the time of acquisition and FE the scaling factor.

For the calculation of errors, the PeakFit numerical analysis option gives the limits of the 95 confidence interval for the estimated values. We subtracted the lower bound from the upper one, dividing the difference by two before averaging the error from both peaks and converting its units to frequency.

$$Erro{r}_{{\upomega }_{B}}=\frac{{S}_{error}+A{S}_{error}}{2}\left(channels\right)\cdot FE\left(\frac{\mathrm{GHz}}{\mathrm{channel}\cdot \mathrm{V}}\right)\cdot ramp)$$

LABVIEW 4 software was used for temperature control and movement of automated table and for GHOST Conection (https://www.ni.com/es-es/support/downloads/software-products/download.labview.html#369643).

OriginLab 6.0 software was used to prepare the graphs (https://www.originlab.com/).

### Statistical analysis

The measured points were classified in regions (left ventricle, interventricular septum, right ventricle and LV-scar) and then averaged. The statistical significance of the differences between group means was later assessed through unpaired Student t-test or one-way ANOVA, as appropriate using GraphPad Prism 7 (https://www.graphpad.com/scientific-software/prism/). In cases where ANOVA indicated inter-region variability, a multiple comparison using Tukey test was performed. Correlation between data obtained using mBS and echocardiography was assed determining Pearson Correlation coefficient. Data are presented as mean ± SD and a p < 0.05 was considered statistically significant.

## Supplementary Information

Supplementary Information.
