# Author Correction: Assessment of myocardial viscoelasticity with Brillouin spectroscopy in myocardial infarction and aortic stenosis models

**DOI:** 10.1038/s41598-021-04261-0

**Published:** 2021-12-23

**Authors:** María Villalba-Orero, Rafael J. Jiménez-Riobóo, Nuria Gontán, Daniel Sanderson, Marina López-Olañeta, Pablo García-Pavía, Manuel Desco, Enrique Lara-Pezzi, Maria Victoria Gómez-Gaviro

**Affiliations:** 1 Centro Nacional de Investigaciones Cardiovasculares (CNIC), Melchor Fernández Almagro, 3, 28029 Madrid, Spain; 2 Instituto de Ciencia de Materiales de Madrid, Consejo Superior de Investigaciones Científicas (CSIC), Madrid, Spain; 3 Instituto de Investigación Sanitaria Gregorio Marañón, Madrid, Spain; 4 Hospital Puerta de Hierro Majadahonda, Madrid, Spain; 5 Centro de Investigación Biomédica en Red Cardiovascular (CIBERCV), Madrid, Spain; 6 Universidad Francisco de Vitoria (UFV), Pozuelo de Alarcon, Spain; 7 Centro de Investigación Biomédica en Red Salud Mental (CIBERSAM), Madrid, Spain; 8 Departamento de Bioingeniería e Ingeniería Aeroespacial, Universidad Carlos III, Madrid, Spain; 9 Hospital General Universitario Gregorio Marañón, Doctor Esquerdo 46, 28007 Madrid, Spain

Correction to: *Scientific Reports* 10.1038/s41598-021-00661-4, published online 01 November 2021

The Acknowledgements section in the original version of this Article was omitted. The Acknowledgements section now reads:

“This study has been funded by Instituto de Salud Carlos III through the project PI18/00462 to M.V.G.G., co‐funded by European Regional Development Fund "A way to make Europe, (CB16/11/00432 to P.G.-P. and E.L-P, and RD12/0042/005) and the Spanish Ministerio de Ciencia (RTI2018-096961-B-I00 to E.L-P. and RTI2018-096918-B-C41 to R.J.J.R.). This study was also supported by the Plan Estatal de I+D+I 2013-2016, with funding from the European Regional Development Fund (ERDF) “A way to build Europe” initiative. The CNIC is supported by Instituto de Salud Carlos III (ISCIII), Ministerio de Ciencia e Innovación (MCIN) and the Pro CNIC Foundation and is a Severo Ochoa Center of Excellence (SEV-2015-0505).”

The original Article has been corrected.

