# Supplementary material for: Assessment of myocardial viscoelasticity with Brillouin spectroscopy in myocardial infarction and aortic stenosis models
Source: Sci Rep. 2021 Nov 1;11:21369. doi: 10.1038/s41598-021-00661-4 (PMC8560820; doi:10.1038/s41598-021-00661-4)
Supplement: Supplementary file 1 — Supplementary Information. [file 41598_2021_661_MOESM1_ESM.docx]

**SUPPLEMENTAL MATERIAL**

**Supplemental Figures and Figure Legends**


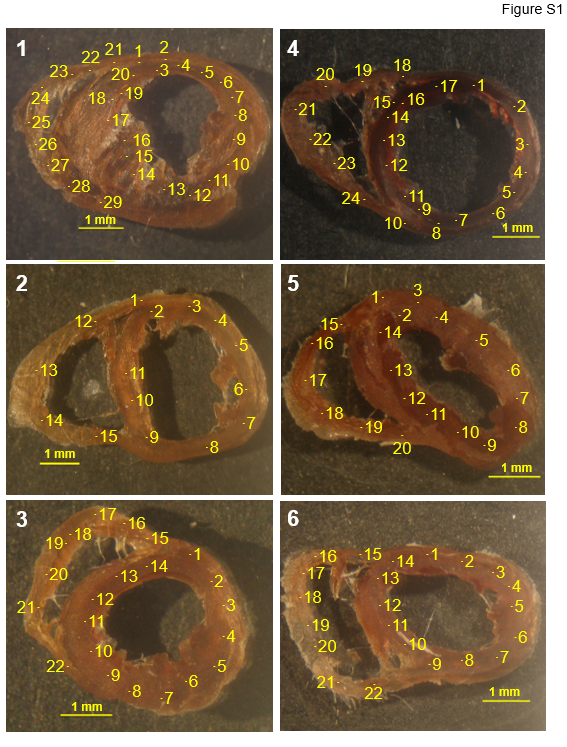


**Figure S1. Points measured by Brillouin microspectroscopy in control uninjured hearts.** Hearts were isolated from adult mice, sectioned, dried, and stored at 4ºC before assessing them with mBS. A total of 132 points were measured. Frequency shift and peak linewidth value obtained for each point are shown in tables S1-S6.


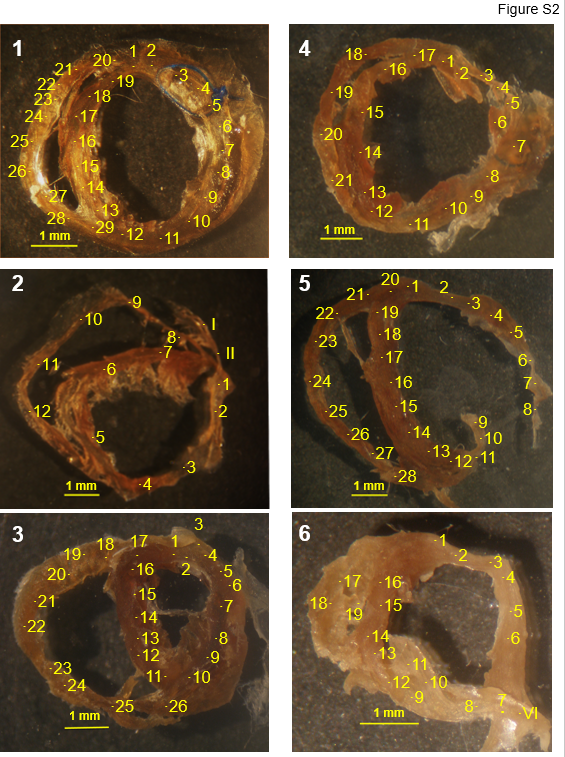


**Figure S2. Points measured by Brillouin microspectroscopy in infarcted hearts.** Myocardial infarction was performed by left anterior descendent coronary artery ligation. Mice were sacrificed 28 days later, and hearts were excised and sliced. Sections were dried and stored at 4ºC until assessed by mBS. Frequency shift and peak linewidth value obtained for each point are shown in tables S7-S12.


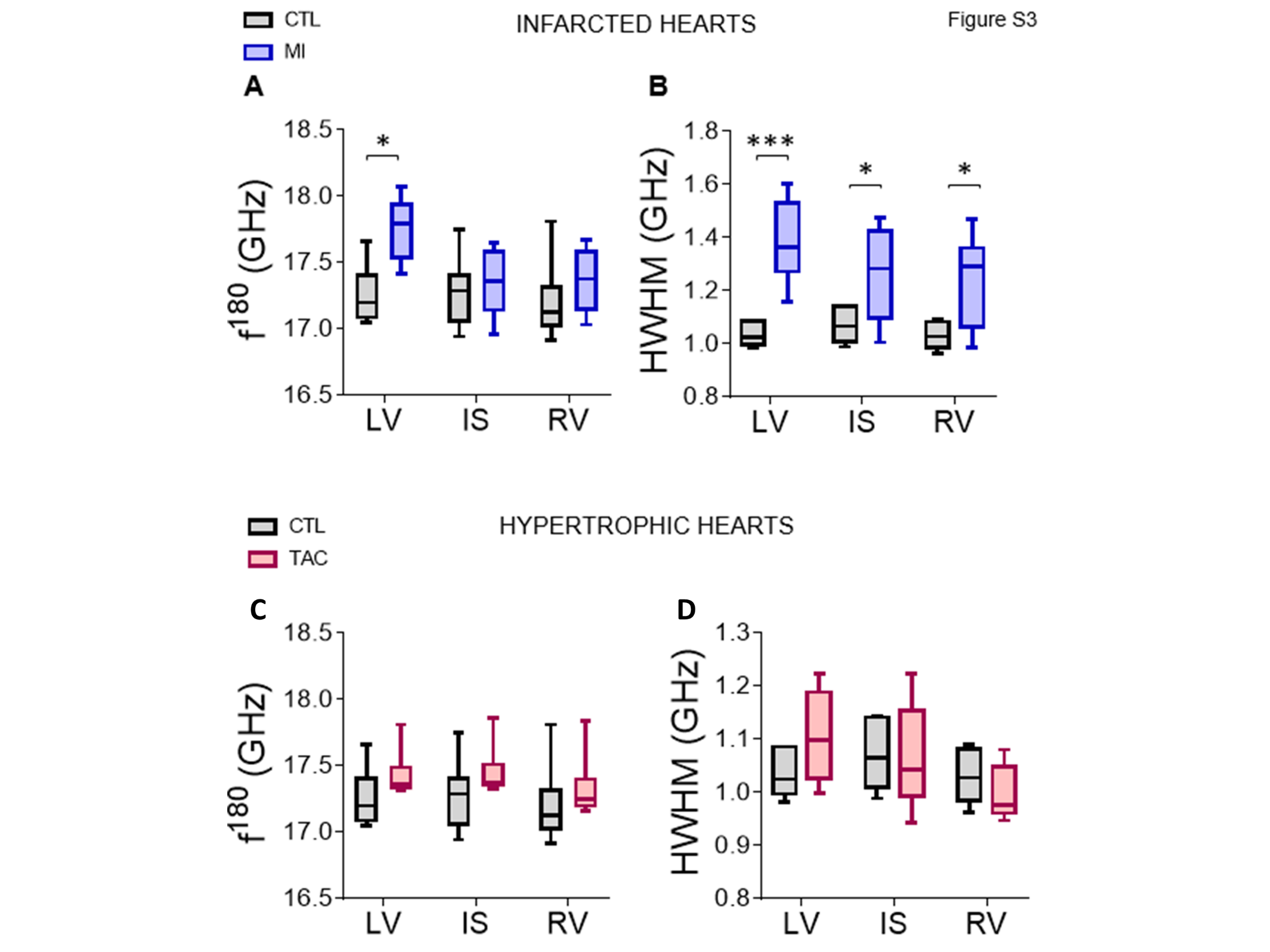


**Figure S3. The damaged myocardium shows a shift in Brillouin frequency. A, B**, Myocardial infarction was induced by permanently ligating the left descending coronary artery. Mice were sacrificed 28 days later and mBS frequency shift (A) and peak width (B) were measured in cardiac sections of infarcted (MI) and uninjured control (CTL) mice. In these graphs, LV encompasses both the infarcted and non-infarcted regions in the left ventricle. **C, D**, Pressure overload was induced by transaortic constriction and mice were sacrificed 21 days later. mBS frequency shift (C) and peak width (D) were measured in cardiac sections of hypertrophic (TAC) and uninjured control (CTL) mice**.** *p< 0.05; ***p< 0.001. 2-way ANOVA and Tukey post-hoc test (n=6 in each segment). LV, left ventricle; IS, interventricular septum; RV, right ventricle.


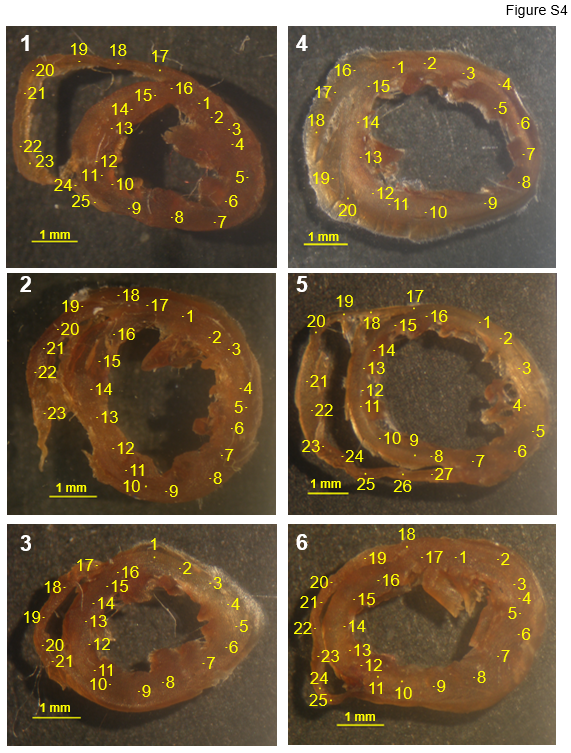


**Figure S4. Points measured by Brillouin microspectroscopy in hypertrophic hearts.** Pressure overload cardiac hypertrophy was induced by transaortic constriction and mice were sacrificed 21 days later. Hearts were excised, sliced, and sections were dried and stored at 4ºC until assessed by mBS. Frequency shift and peak linewidth value obtained for each point are shown in table S13-S18.

**Supplemental Tables**

Table S1. Sample Ctl_1. Data for f^180^, its error and for HWHM obtained in each point drawn in figure S1.1.

|  | | **LV** | | | **SEPTUM** | | | | **RV** | | | |
| --- | --- | --- | --- | --- | --- | --- | --- | --- | --- | --- | --- | --- |
| **Point** | **f^180^**  **(GHz)** | | **error** | **HWHM**  **(GHz)** | **Point** | **f^180^**  **(GHz)** | **error** | **HWHM**  **(GHz)** | **Point** | **f^180^**  **(GHz)** | **error** | **HWHM**  **(GHz)** |
| **3** | 17.38 | | 0.05 | 1.07 | **14** | 17.84 | 0.05 | 1.05 | **21** | 17.85 | 0.03 | 0.99 |
| **2** | 17.86 | | 0.03 | 0.91 | **15** | 17.70 | 0.05 | 0.96 | **22** | 17.85 | 0.13 | 1.10 |
| **1** | 17.79 | | 0.03 | 0.97 | **16** | 17.82 | 0.04 | 0.96 | **23** | 17.79 | 0.13 | 1.40 |
| **4** | 17.50 | | 0.05 | 1.01 | **17** | 17.59 | 0.04 | 1.08 | **24** | 17.77 | 0.05 | 0.92 |
| **5** | 17.58 | | 0.03 | 1.01 | **18** | 17.75 | 0.03 | 0.83 | **25** | 17.76 | 0.03 | 0.89 |
| **6** | 17.48 | | 0.03 | 1.12 | **19** | 17.70 | 0.04 | 1.02 | **26** | 17.70 | 0.03 | 0.96 |
| **7** | 17.56 | | 0.03 | 1.10 | **20** | 17.79 | 0.05 | 1.01 | **27** | 17.89 | 0.03 | 0.85 |
| **8** | 17.74 | | 0.03 | 0.96 |  |  |  |  | **28** | 17.77 | 0.04 | 1.14 |
| **9** | 17.63 | | 0.03 | 1.03 |  |  |  |  | **29** | 17.87 | 0.03 | 0.97 |
| **10** | 17.73 | | 0.03 | 0.98 |  |  |  |  |  |  |  |  |
| **11** | 17.78 | | 0.04 | 0.94 |  |  |  |  |  |  |  |  |
| **12** | 17.84 | | 0.04 | 0.99 |  |  |  |  |  |  |  |  |
| **13** | 17.72 | | 0.05 | 1.08 |  |  |  |  |  |  |  |  |

Table S2. Sample Ctl_2. Data for f^180^, its error and for HWHM obtained in each point drawn in figure S1.2.

| **LV** | | | | **SEPTUM** | | |  | **RV** | | |  |
| --- | --- | --- | --- | --- | --- | --- | --- | --- | --- | --- | --- |
| **Point** | **f^180^**  **(GHz)** | **error** | **HWHM**  **(GHz)** | **Point** | **f^180^**  **(GHz)** | **error** | **HWHM**  **(GHz)** | **Point** | **f^180^**  **(GHz)** | **error** | **HWHM**  **(GHz)** |
| **2** | 17.29 | 0.04 | 1.05 | **9** | 17.13 | 0.09 | 1.28 | **12** | 17.21 | 0.06 | 0.78 |
| **1** | 17.32 | 0.05 | 1.07 | **10** | 17.31 | 0.06 | 1.01 | **13** | 17.09 | 0.05 | 1.02 |
| **3** | 17.31 | 0.08 | 1.10 | **11** | 17.33 | 0.06 | 1.14 | **14** | 17.22 | 0.05 | 0.98 |
| **4** | 17.28 | 0.08 | 1.10 |  |  | 0.08 | 1.23 | **15** | 17.09 | 0.10 | 1.20 |
| **5** | 17.26 | 0.08 | 0.98 |  |  | 0.05 | 1.00 |  |  |  |  |
| **6** | 17.46 | 0.07 | 0.98 |  |  |  |  |  |  |  |  |
| **7** | 17.56 | 0.08 | 1.23 |  |  |  |  |  |  |  |  |
| **8** | 17.20 | 0.03 | 1.15 |  |  |  |  |  |  |  |  |

Table S3. Sample Ctl_3. Data for f^180^, its error and for HWHM obtained in each point drawn in figure S1.3.

| **LV** | | | | **SEPTUM** | | | | **RV** | | | |
| --- | --- | --- | --- | --- | --- | --- | --- | --- | --- | --- | --- |
| **Point** | **f^180^**  **(GHz)** | **error** | **HWHM**  **(GHz)** | **Point** | **f^180^**  **(GHz)** | **error** | **HWHM**  **(GHz)** | **Point** | **f^180^**  **(GHz)** | **error** | **HWHM**  **(GHz)** |
| **1** | 17.40 | 0.04 | 0.93 | **11** | 17.42 | 0.06 | 0.98 | **22** | 17.16 | 0.07 | 0.92 |
| **2** | 17.21 | 0.06 | 0.85 | **12** | 17.25 | 0.14 | 1.15 | **21** | 17.20 | 0.05 | 0.99 |
| **3** | 17.05 | 0.12 | 1.29 | **13** | 17.20 | 0.12 | 1.05 | **20** | 17.09 | 0.05 | 1.01 |
| **4** | 17.12 | 0.03 | 0.84 | **14** | 17.30 | 0.05 | 1.13 | **19** | 17.06 | 0.04 | 1.10 |
| **5** | 17.14 | 0.06 | 1.03 |  |  |  |  | **18** | 17.19 | 0.04 | 0.99 |
| **6** | 17.27 | 0.06 | 0.97 |  |  |  |  | **17** | 17.26 | 0.05 | 0.85 |
| **7** | 17.19 | 0.07 | 0.99 |  |  |  |  | **16** | 17.15 | 0.08 | 0.85 |
| **8** | 17.28 | 0.05 | 0.88 |  |  |  |  | **15** | 17.21 | 0.05 | 0.99 |
| **9** | 17.20 | 0.08 | 0.98 |  |  |  |  |  |  |  |  |
| **10** | 17.14 | 0.04 | 1.05 |  |  |  |  |  |  |  |  |

Table S4. Sample Ctl_4. Data for f^180^, its error and for HWHM obtained in each point drawn in figure S1.4.

| **LV** | | | | **SEPTUM** | | | | **RV** | | | |
| --- | --- | --- | --- | --- | --- | --- | --- | --- | --- | --- | --- |
| **Point** | **f^180^**  **(GHz)** | **error** | **HWHM**  **(GHz)** | **Point** | **f^180^**  **(GHz)** | **error** | **HWHM**  **(GHz)** | **Point** | **f^180^**  **(GHz)** | **error** | **HWHM**  **(GHz)** |
| **1** | 17.30 | 0.03 | 1.07 | **12** | 17.18 | 0.03 | 1.04 | **18** | 17.06 | 0.03 | 1.15 |
| **2** | 17.23 | 0.03 | 1.11 | **13** | 17.16 | 0.03 | 1.06 | **19** | 17.15 | 0.03 | 1.08 |
| **3** | 17.20 | 0.03 | 1.07 | **14** | 17.34 | 0.025 | 0.89 | **20** | 16.99 | 0.03 | 0.92 |
| **4** | 17.19 | 0.02 | 0.86 | **16** | 17.32 | 0.03 | 1.04 | **21** | 17.02 | 0.03 | 0.98 |
| **5** | 17.20 | 0.02 | 0.98 | **15** | 17.33 | 0.03 | 0.89 | **22** | 17.06 | 0.03 | 0.96 |
| **6** | 17.26 | 0.02 | 0.93 | **17** | 17.36 | 0.03 | 1.07 | **23** | 17.23 | 0.03 | 0.97 |
| **7** | 17.22 | 0.02 | 0.99 | **11** | 17.13 | 0.03 | 1.00 | **24** | 17.18 | 0.03 | 0.87 |
| **8** | 17.05 | 0.02 | 0.97 |  |  |  |  | **10** | 17.36 | 0.03 | 0.93 |
| **9** | 17.05 | 0.03 | 1.04 |  |  |  |  |  |  |  |  |

Table S5. Sample Ctl_5. Data for f^180^, its error and for HWHM obtained in each point drawn in figure S1.5.

| **LV** | | | | **SEPTUM** | | | | **RV** | | | |
| --- | --- | --- | --- | --- | --- | --- | --- | --- | --- | --- | --- |
| **Point** | **f^180^**  **(GHz)** | **ν^B^_error_** | **HWHM**  **(GHz)** | **Point** | **f^180^**  **(GHz)** | **ν^B^_error_** | **HWHM**  **(GHz)** | **Point** | **f^180^**  **(GHz)** | **ν^B^_error_** | **HWHM**  **(GHz)** |
| **4** | 17.01 | 0.03 | 1.03 | **11** | 16.98 | 0.03 | 1.12 | **15** | 16.95 | 0.03 | 1.20 |
| **5** | 17.12 | 0.03 | 0.98 | **12** | 16.96 | 0.03 | 1.04 | **16** | 17.07 | 0.03 | 1.11 |
| **6** | 17.12 | 0.03 | 0.98 | **13** | 16.97 | 0.03 | 1.19 | **17** | 16.93 | 0.03 | 1.03 |
| **7** | 17.13 | 0.03 | 1.05 | **14** | 16.86 | 0.03 | 1.18 | **18** | 16.85 | 0.03 | 1.06 |
| **8** | 17.11 | 0.03 | 1.11 | **2** | 16.95 | 0.03 | 1.151 | **19** | 16.75 | 0.03 | 1.03 |
| **9** | 17.00 | 0.03 | 0.99 |  |  |  |  | **20** | 16.89 | 0.03 | 1.15 |
| **10** | 17.00 | 0.03 | 0.97 |  |  |  |  | **1** | 16.98 | 0.03 | 1.1 |
| **3** | 17.04 | 0.03 | 1.09 |  |  |  |  |  |  |  |  |

Table S6. Sample Ctl_6. Data for f^180^, its error and for HWHM obtained in each point drawn in figure S1.6.

| **LV** | | | | **SEPTUM** | | | | **RV** | | | |
| --- | --- | --- | --- | --- | --- | --- | --- | --- | --- | --- | --- |
| **Point** | **f^180^**  **(GHz)** | **error** | **HWHM**  **(GHz)** | **Point** | **f^180^**  **(GHz)** | **error** | **HWHM**  **(GHz)** | **Point** | **f^180^**  **(GHz)** | **error** | **HWHM**  **(GHz)** |
| **4** | 16.96 | 0.03 | 1.17 | **10** | 17.07 | 0.03 | 1.16 | **15** | 16.88 | 0.03 | 1.00 |
| **5** | 17.12 | 0.03 | 1.11 | **11** | 16.95 | 0.03 | 1.02 | **16** | 16.81 | 0.04 | 1.30 |
| **6** | 17.01 | 0.035 | 1.08 | **12** | 17.10 | 0.03 | 1.06 | **17** | 17.03 | 0.03 | 0.98 |
| **7** | 17.12 | 0.035 | 1.06 | **13** | 17.12 | 0.02 | 1.00 | **18** | 17.27 | 0.03 | 0.97 |
| **8** | 17.17 | 0.03 | 1.01 | **14** | 17.14 | 0.035 | 1.14 | **19** | 17.18 | 0.03 | 0.97 |
| **9** | 17.05 | 0.03 | 1.24 |  |  |  |  | **20** | 17.12 | 0.03 | 1.09 |
| **1** | 17.10 | 0.03 | 1.01 |  |  |  |  | **21** | 17.02 | 0.035 | 1.24 |
| **2** | 17.10 | 0.03 | 1.12 |  |  |  |  | **22** | 17.00 | 0.035 | 1.16 |
| **3** | 17.14 | 0.03 | 1.03 |  |  |  |  |  |  |  |  |

Table S7. Sample MI_1. Data for f^180^, its error and for HWHM obtained in each point drawn in figure S2.1.

| **LV** | | | | **SEPTUM** | | | | **RV** | | | |
| --- | --- | --- | --- | --- | --- | --- | --- | --- | --- | --- | --- |
| **Point** | **f^180^**  **(GHz)** | **error** | **HWWM**  **(GHz)** | **Point** | **f^180^**  **(GHz)** | **error** | **HWHM**  **(GHz)** | **Point** | **f^180^**  **(GHz)** | **error** | **HWHM**  **(GHz)** |
| **1** | 18.06 | 0.10 | 1.38 | **13** | 17.43 | 0.07 | 0.90 | **20** | 17.85 | 0.06 | 0.92 |
| **2** | 18.34 | 0.06 | 1.17 | **14** | 17.50 | 0.05 | 0.87 | **21** | 17.79 | 0.12 | 1.17 |
| **3** | 18.19 | 0.11 | 1.75 | **15** | 17.43 | 0.06 | 0.91 | **22** | 17.74 | 0.11 | 1.25 |
| **4** | 17.73 | 0.10 | 1.08 | **16** | 17.43 | 0.09 | 1.13 | **23** | 17.45 | 0.07 | 0.85 |
| **5** | 17.85 | 0.08 | 1.11 | **17** | 17.48 | 0.08 | 1.03 | **24** | 17.47 | 0.06 | 0.95 |
| **6** | 18.23 | 0.07 | 1.26 | **18** | 17.58 | 0.09 | 0.9 | **25** | 17.47 | 0.06 | 0.84 |
| **7** | 17.82 | 0.03 | 0.99 | **19** | 18.19 | 0.08 | 1.29 | **26** | 17.45 | 0.07 | 0.85 |
| **8** | 17.52 | 0.06 | 0.91 |  |  |  |  | **27** | 17.55 | 0.07 | 0.88 |
| **9** | 17.47 | 0.07 | 0.95 |  |  |  |  | **28** | 17.42 | 0.08 | 1.09 |
| **10** | 17.44 | 0.04 | 0.97 |  |  |  |  | **29** | 17.52 | 0.07 | 0.88 |
| **11** | 17.46 | 0.07 | 1.05 |  |  |  |  |  |  |  |  |
| **12** | 17.43 | 0.06 | 0.90 |  |  |  |  |  |  |  |  |

Table S8. Sample MI_2. Data for f^180^, its error and for HWHM obtained in each point drawn in figure S2.2.

| **LV** | | | | **SEPTUM** | | | | **RV** | | | |
| --- | --- | --- | --- | --- | --- | --- | --- | --- | --- | --- | --- |
| **Point** | **f^180^**  **(GHz)** | **error** | **HWHM**  **(GHz)** | **Point** | **f^180^**  **(GHz)** | **error** | **HWHM**  **(GHz)** | **Point** | **f^180^**  **(GHz)** | **error** | **HWHM**  **(GHz)** |
| **1** | 18.50 | 0.08 | 1.42 | **5** | 17.08 | 0.06 | 1.01 | **12** | 17.03 | 0.05 | 1.31 |
| **2** | 17.45 | 0.05 | 1.45 | **6** | 16.69 | 0.13 | 1.35 | **11** | 17.01 | 0.06 | 1.24 |
| **3** | 17.98 | 0.08 | 1.166 | **7** | 17.08 | 0.01 | 1.32 | **10** | 16.93 | 0.05 | 1.29 |
| **4** | 17.45 | 0.044 | 1.88 |  |  |  |  | **9** | 16.99 | 0.05 | 1.4 |
|  |  |  |  |  |  |  |  | **8** | 17.18 | 0.07 | 1.33 |

Table S9. Sample MI_3. Data for f^180^, its error and for HWHM obtained in each point drawn in figure S2.3.

| **LV** | | | | **SEPTUM** | | | | **RV** | | | |
| --- | --- | --- | --- | --- | --- | --- | --- | --- | --- | --- | --- |
| **Point** | **f^180^**  **(GHz)** | **error** | **HWHM**  **(GHz)** | **Point** | **f^180^**  **(GHz)** | **error** | **HWHM**  **(GHz)** | **Point** | **f^180^**  **(GHz)** | **error** | **HWHM**  **(GHz)** |
| **1** | 17.68 | 0.06 | 1.85 | **11** | 17.70 | 0.55 | 1.11 | **18** | 17.61 | 0.04 | 1.14 |
| **2** | 18.33 | 0.07 | 1.44 | **12** | 17.52 | 0.045 | 1.07 | **19** | 17.75 | 0.07 | 1.09 |
| **3** | 17.73 | 0.07 | 1.51 | **13** | 17.67 | 0.41 | 1.33 | **20** | 17.87 | 0.04 | 1.05 |
| **4** | 18.09 | 0.06 | 1.23 | **14** | 17.71 | 0.50 | 1.18 | **21** | 17.64 | 0.03 | 1.12 |
| **5** | 18.23 | 0.08 | 1.61 | **15** | 17.68 | 0.04 | 1.07 | **22** | 17.48 | 0.04 | 1.08 |
| **6** | 18.60 | 0.08 | 1.19 | **16** | 17.62 | 0.03 | 1.00 | **23** | 17.67 | 0.051 | 1.06 |
| **7** | 17.57 | 0.05 | 1.04 |  |  |  |  | **24** | 17.67 | 0.056 | 1.08 |
| **8** | 17.68 | 0.04 | 1.02 |  |  |  |  | **25** | 17.68 | 0.08 | 0.89 |
| **9** | 17.80 | 0.38 | 1.02 |  |  |  |  | **26** | 17.63 | 0.71 | 1.30 |
| **10** | 17.70 | 0.06 | 1.01 |  |  |  |  |  |  |  |  |
| **17** | 17.59 | 0.069 | 1.29 |  |  |  |  |  |  |  |  |

Table S10. Sample MI_4. Data for f^180^, its error and for HWHM obtained in each point drawn in figure S2.4.

| **LV** | | | | **SEPTUM** | | | | **RV** | | | |
| --- | --- | --- | --- | --- | --- | --- | --- | --- | --- | --- | --- |
| **Point** | **f^180^**  **(GHz)** | **error** | **HWHM**  **(GHz)** | **Point** | **f^180^**  **(GHz)** | **error** | **HWHM**  **(GHz)** | **Point** | **f^180^**  **(GHz)** | **error** | **HWHM**  **(GHz)** |
| **1** | 17.17 | 0.09 | 1.63 | **1** | 17.39 | 0.09 | 1.49 | **18** | 17.21 | 0.07 | 1.23 |
| **2** | 17.23 | 0.083 | 1.49 | **13** | 17.45 | 0.06 | 1.24 | **19** | 17.25 | 0.12 | 1.85 |
| **3** | 17.12 | 0.078 | 1.30 | **14** | 17.39 | 0.08 | 1.07 | **20** | 17.36 | 0.08 | 1.29 |
| **4** | 17.28 | 0.08 | 1.66 | **15** | 17.56 | 0.06 | 1.17 | **21** | 17.38 | 0.09 | 1.49 |
| **5** | 17.24 | 0.19 | 1.51 | **16** | 17.32 | 0.07 | 1.69 |  |  |  |  |
| **6** | 17.25 | 0.06 | 1.45 | **17** | 17.36 | 0.10 | 1.78 |  |  |  |  |
| **7** | 17.41 | 0.15 | 1.87 |  |  |  |  |  |  |  |  |
| **8** | 18.71 | 0.10 | 1.42 |  |  |  |  |  |  |  |  |
| **9** | 18.62 | 0.36 | 1.29 |  |  |  |  |  |  |  |  |
| **10** | 17.69 | 0.06 | 1.39 |  |  |  |  |  |  |  |  |
| **11** | 17.45 | 0.08 | 1.53 |  |  |  |  |  |  |  |  |

Table S11. Sample MI_5. Data for f^180^, its error and for HWHM obtained in each point drawn in figure S2.5.

| **LV** | | | | **SEPTUM** | | | | **RV** | | | |
| --- | --- | --- | --- | --- | --- | --- | --- | --- | --- | --- | --- |
| **Point** | **f^180^**  **(GHz)** | **error** | **HWHM**  **(GHz)** | **Point** | **f^180^**  **(GHz)** | **error** | **HWHM**  **(GHz)** | **Point** | **fν^180^**  **(GHz)** | **error** | **HWHM**  **(GHz)** |
| **1** | 17.18 | 0..08 | 1.44 | **13** | 17.41 | 0.06 | 1.45 | **28** | 17.59 | 0.08 | 1.02 |
| **2** | 17.68 | 0.09 | 1.74 | **14** | 17.07 | 0.08 | 1.48 | **27** | 17.40 | 0.09 | 1.20 |
| **3** | 18.16 | 0.08 | 1.91 | **15** | 17.20 | 0.05 | 1.39 | **26** | 17.32 | 0.06 | 1.20 |
| **4** | 17.75 | 0.10 | 1.55 | **16** | 17.13 | 0.06 | 1.26 | **25** | 17.48 | 0.15 | 1.71 |
| **5** | 17.72 | 0.09 | 2.01 | **17** | 17.06 | 0.06 | 1.43 | **24** | 17.54 | 0.08 | 1.28 |
| **6** | 18.73 | 0.0 | 0.97 | **18** | 17.08 | 0.07 | 1.53 | **23** | 17.38 | 0.18 | 1.44 |
| **7** | 18.77 | 0.07 | 1.31 | **19** | 17.29 | 0.10 | 1.58 | **22** | 17.48 | 0.07 | 1.48 |
| **8** | 18.13 | 0.06 | 1.33 | **20** | 17.19 | 0.08 | 1.67 | **21** | 17.42 | 0.10 | 1.26 |
| **9** | 18.71 | 0.08 | 1.68 |  |  |  |  |  |  |  |  |
| **10** | 18.66 | 0.09 | 1.56 |  |  |  |  |  |  |  |  |
| **11** | 17.92 | 0.10 | 1.76 |  |  |  |  |  |  |  |  |
| **12** | 17.48 | 0.12 | 1.94 |  |  |  |  |  |  |  |  |

Table S12. Sample MI_6. Data for f^180^, its error and for HWHM obtained in each point drawn in figure S2.6.

| **LV** | | | | **SEPTUM** | | | | **RV** | | | |
| --- | --- | --- | --- | --- | --- | --- | --- | --- | --- | --- | --- |
| **Point** | **f^180^**  **(GHz)** | **error** | **HWHM**  **(GHz)** | **Point** | **f^180^**  **(GHz)** | **error** | **HWHM**  **(GHz)** | **Point** | **f^180^**  **(GHz)** | **error** | **HWHM**  **(GHz)** |
| **2** | 17.80 | 0.045 | 1.24 | **13** | 17.24 | 0.03 | 1.44 | **19** | 17.37 | 0.06 | 1.07 |
| **3** | 17.93 | 0.04 | 1.38 | **14** | 17.35 | 0.42 | 1.39 | **18** | 17.19 | 0.50 | 1.29 |
| **4** | 17.82 | 0.042 | 1.42 | **15** | 17.50 | 0.07 | 1.54 | **17** | 16.95 | 0.08 | 1.44 |
| **1** | 17.50 | 0.07 | 1.09 | **16** | 17.16 | 0.04 | 0.945 |  |  |  |  |
| **5** | 17.42 | 0.05 | 1.26 |  |  |  |  |  |  |  |  |
| **6** | 16.75 | 0.059 | 1.77 |  |  |  |  |  |  |  |  |
| **7** | 17.56 | 0.03 | 1.51 |  |  |  |  |  |  |  |  |
| **8** | 17.06 | 0.04 | 1.69 |  |  |  |  |  |  |  |  |
| **9** | 17.45 | 0.049 | 1.36 |  |  |  |  |  |  |  |  |
| **10** | 17.11 | 0.46 | 1.40 |  |  |  |  |  |  |  |  |
| **11** | 17.55 | 0.09 | 1.38 |  |  |  |  |  |  |  |  |
| **12** | 17.20 | 0.09 |  |  |  |  |  |  |  |  |  |

Table S13. Sample TAC_1. Data for f^180^, its error and for HWHM obtained in each point drawn in figure S4.1.

| LV | | | | SEPTUM | | | | RV | | | |
| --- | --- | --- | --- | --- | --- | --- | --- | --- | --- | --- | --- |
| **Point** | **f^180^**  **(GHz)** | **error** | **HWHM**  **(GHz)** | **Point** | **f^180^**  **(GHz)** | **error** | **HEHM**  **(GHz)** | **Point** | **f^180^**  **(GHz)** | **error** | **HWHM**  **(GHz)** |
| **1** | 17.36 | 0.06 | 0.94 | **10** | 17.41 | 0.06 | 1.07 | **24** | 17.27 | 0.04 | 1.01 |
| **2** | 17.42 | 0.07 | 0.98 | **11** | 17.31 | 0.06 | 1.18 | **25** | 16.90 | 0.15 | 1.56 |
| **3** | 17.42 | 0.05 | 0.94 | **12** | 17.40 | 0.06 | 1.09 | **17** | 17.21 | 0.07 | 0.79 |
| **4** | 17.33 | 0.07 | 1.12 | **13** | 17.39 | 0.03 | 1.08 | **18** | 17.37 | 0.03 | 0.77 |
| **5** | 17.41 | 0.08 | 1.00 | **14** | 17.35 | 0.05 | 1.04 | **19** | 17.30 | 0.05 | 0.98 |
| **6** | 17.36 | 0.06 | 1.08 | **15** | 17.55 | 0.05 | 0.92 | **20** | 17.18 | 0.06 | 1.09 |
| **7** | 17.29 | 0.03 | 0.97 | **16** | 17.44 | 0.08 | 0.91 | **21** | 17.13 | 0.03 | 0.95 |
| **8** | 17.26 | 0.03 | 0.96 |  |  |  |  | **22** | 17.01 | 0.04 | 0.88 |
| **9** | 17.34 | 0.04 | 0.99 |  |  |  |  | **23** | 17.10 | 0.06 | 0.76 |

|  |  |  |
| --- | --- | --- |

Table S14. Sample TAC_2. Data for f^180^, its error and for HWHM obtained in each point drawn in figure S4.2.

| LV | | | | SEPTUM | | | | RV | | | |
| --- | --- | --- | --- | --- | --- | --- | --- | --- | --- | --- | --- |
| **Point** | **f^180^**  **(GHz)** | **error** | **HWHM**  **(GHz)** | **Point** | **f^180^**  **(GHz)** | **error** | **HWHM**  **(GHz)** | **Point** | **f^180^**  **(GHz)** | **error** | **HWHM**  **(GHz)** |
| **1** | 18.00 | 0.09 | 1.21 | **11** | 17.94 | 0.043 | 1.07 | **18** | 17.85 | 0.05 | 1.27 |
| **2** | 17.78 | 0.09 | 1.51 | **12** | 17.77 | 0.069 | 1.13 | **19** | 17.75 | 0.04 | 1.02 |
| **3** | 17.72 | 0.07 | 1.22 | **13** | 17.73 | 0.059 | 1.19 | **20** | 17.86 | 0.06 | 1.09 |
| **4** | 17.85 | 0.08 | 1.26 | **14** | 17.86 | 0.06 | 1.09 | **21** | 17.73 | 0.04 | 1.08 |
| **5** | 17.78 | 0.11 | 1.04 | **15** | 17.89 | 0.06 | 1.33 | **22** | 17.95 | 0.06 | 1.15 |
| **6** | 17.75 | 0.05 | 1.22 | **16** | 18.03 | 007 | 1.47 | **23** | 17.88 | 0.06 | 0.88 |
| **7** | 17.79 | 0.06 | 1.19 | **17** | 17.87 | 0.03 | 0.95 |  |  |  |  |
| **8** | 17.86 | 0.05 | 1.09 |  |  |  |  |  |  |  |  |
| **9** | 17.61 | 0.07 | 1.38 |  |  |  |  |  |  |  |  |
| **10** | 17.88 | 0.06 | 1.13 |  |  |  |  |  |  |  |  |

Table S15. Sample TAC_3. Data for f^180^, its error and for HWHM obtained in each point drawn in figure S4.3.

| LV | | | | SEPTUM | | | | RV | | | |
| --- | --- | --- | --- | --- | --- | --- | --- | --- | --- | --- | --- |
| **Point** | **f^180^**  **(GHz)** | **error** | **HWHM**  **(GHz)** | **Point** | **f^180^**  **(GHz)** | **error** | **HWHM**  **(GHz)** | **Point** | **f^180^**  **(GHz)** | **error** | **HWHM**  **(GHz)** |
| **1** | 17.37 | 0.10 | 1.08 | **11** | 17.31 | 0.06 | 1.06 | **21** | 17.19 | 0.10 | 1.06 |
| **2** | 17.30 | 0.08 | 0.89 | **12** | 17.36 | 0.06 | 1.11 | **20** | 17.14 | 0.08 | 0.94 |
| **3** | 17.26 | 0.08 | 0.96 | **13** | 17.39 | 0.08 | 1.17 | **19** | 17.20 | 0.09 | 0.97 |
| **4** | 17.16 | 0.07 | 1.01 | **14** | 17.31 | 0.11 | 1.05 | **18** | 17.27 | 0.10 | 0.10 |
| **5** | 17.12 | 0.07 | 1.18 | **15** | 17.35 | 0.07 | 0.72 | **17** | 17.15 | 0.11 | 1.10 |
| **6** | 17.28 | 0.08 | 1.16 | **16** | 17.26 | 0.10 | 1.15 |  |  |  |  |
| **7** | 17.37 | 0.08 | 1.18 |  |  |  |  |  |  |  |  |
| **8** | 17.40 | 0.10 | 1.14 |  |  |  |  |  |  |  |  |
| **9** | 17.38 | 0.08 | 0.98 |  |  |  |  |  |  |  |  |
| **10** | 17.41 | 0.08 | 1.00 |  |  |  |  |  |  |  |  |

Table S16. Sample TAC_4. Data for f^180^, its error and for HWHM obtained in each point drawn in figure S4.4.

| LV | | | | SEPTUM | | | | RV | | | |
| --- | --- | --- | --- | --- | --- | --- | --- | --- | --- | --- | --- |
| **Point** | **f^180^**  **(GHz)** | **error** | **HWHM**  **(GHz)** | **Point** | **f^180^**  **(GHz)** | **error** | **HWHM**  **(GHz)** | **Point** | **f^180^**  **(GHz)** | **error** | **HWHM**  **(GHz)** |
| **1** | 17.37 | 0.93 | 0.15 | **11** | 17.24 | 0.77 | 0.14 | **20** | 17.27 | 0.95 | 0.15 |
| **2** | 17.26 | 1.24 | 1.01 | **12** | 17.24 | 0.95 | 0.99 | **19** | 17.28 | 0.95 | 0.14 |
| **3** | 17.32 | 1.06 | 1.00 | **13** | 17.40 | 0.87 | 0.10 | **18** | 17.31 | 0.949 | 0.13 |
| **4** | 17.40 | 0.97 | 1.00 | **14** | 17.39 | 1.11 | 0.13 | **17** | 17.16 | 0.94 | 0.12 |
| **5** | 17.44 | 1.49 | 1.01 | **15** | 17.40 | 1.00 | 0.19 | **16** | 17.34 | 0.95 | 0.13 |
| **6** | 17.47 | 1.15 | 1.04 |  |  |  |  |  |  |  |  |
| **7** | 17.31 | 1.10 | 0.05 |  |  |  |  |  |  |  |  |
| **8** | 17.60 | 1.43 | 1.03 |  |  |  |  |  |  |  |  |
| **9** | 17.33 | 1.02 | 0.19 |  |  |  |  |  |  |  |  |
| **10** | 17.44 | 1.14 | 0.09 |  |  |  |  |  |  |  |  |

Table S17. Sample TAC_5. Data for f^180^, its error and for HWHM obtained in each point drawn in figure S4.5.

| **LV** | | | | **SEPTUM** | | | | **RV** | | | |
| --- | --- | --- | --- | --- | --- | --- | --- | --- | --- | --- | --- |
| **Point** | **f^180^**  **(GHz)** | **error** | **HWHM**  **(GHz)** | **Point** | **f^180^**  **(GHz)** | **error** | **HWHM**  **(GHz)** | **Point** | **f^180^**  **(GHz)** | **error** | **HWHM**  **(GHz)** |
| **1** | 17.34 | 0.08 | 1.10 | **8** | 17.39 | 0.08 | 0.92 | **27** | 17.15 | 0.07 | 1.07 |
| **2** | 17.26 | 0.07 | 1.11 | **9** | 17.38 | 0.07 | 1.14 | **26** | 17.18 | 0.06 | 0.10 |
| **3** | 17.07 | 0.08 | 1.24 | **10** | 17.25 | 0.07 | 1.08 | **25** | 17.29 | 0.07 | 0.92 |
| **4** | 17.27 | 0.08 | 1.05 | **11** | 17.32 | 0.05 | 1.00 | **24** | 17.06 | 0.11 | 1.21 |
| **5** | 17.39 | 0.07 | 1.12 | **12** | 17.33 | 0.06 | 1.10 | **23** | 17.29 | 0.04 | 0.97 |
| **6** | 17.36 | 0.06 | 1.10 | **13** | 17.43 | 0.10 | 1.16 | **22** | 17.24 | 0.06 | 0.99 |
| **7** | 17.43 | 0.09 | 1.03 | **14** | 17.58 | 0.06 | 1.08 | **21** | 17.27 | 0.06 | 0.87 |
|  |  |  |  | **15** | 17.37 | 0.07 | 1.20 | **20** | 17.24 | 0.07 | 0.91 |
|  |  |  |  | **16** | 17.50 | 0.05 | 0.99 | **19** | 17.32 | 0.08 | 0.87 |
|  |  |  |  |  |  |  |  | **17** | 17.52 | 0.06 | 1.15 |
|  |  |  |  |  |  |  |  | **18** | 17.39 | 0.07 | 1.06 |

Table S18. Sample TAC_6. Data for f^180^, its error and for HWHM obtained in each point drawn in figure S4.6.

| **LV** | | | | **SEPTUM** | | | | **RV** | | | |
| --- | --- | --- | --- | --- | --- | --- | --- | --- | --- | --- | --- |
| **Point** | **f^180^**  **(GHz)** | **error** | **HWHM**  **(GHz)** | **Point** | **f^180^**  **(GHz)** | **error** | **HWHM**  **(GHz)** | **Point** | **f^180^**  **(GHz)** | **error** | **HWHM(GHz)** |
| **1** | 17.32 | 1.55 | 1.55 | **12** | 17.15 | 1.27 | 1.2713 | **25** | 17.29 | 0.94 | 0.94 |
| **2** | 17.35 | 1.18 | 1.18 | **13** | 17.40 | 0.98 | 0.9816 | **24** | 17.20 | 0.94 | 0.94 |
| **3** | 17.37 | 1.21 | 1.21 | **14** | 17.32 | 1.11 | 1.1136 | **23** | 17.28 | 1.23 | 1.23 |
| **5** | 17.36 | 1.14 | 1.15 | **15** | 17.53 | 1.21 | 1.2143 | **22** | 17.29 | 1.42 | 1.42 |
| **4** | 17.11 | 1.15 | 1.15 | **16** | 17.50 | 1.10 | 1.1036 | **21** | 17.31 | 1.20 | 1.20 |
| **6** | 17.28 | 1.02 | 1.02 | **17** | 17.39 | 1.37 | 1.3768 | **20** | 17.38 | 1.00 | 1.00 |
| **7** | 17.32 | 1.41 | 1.41 |  |  |  |  | **19** | 17.20 | 1.10 | 1.10 |
| **8** | 17.34 | 1.21 | 1.21 |  |  |  |  | **18** | 17.24 | 1.02 | 1.03 |
| **9** | 17.39 | 1.16 | 1.17 |  |  |  |  |  |  |  |  |
| **10** | 17.28 | 1.05 | 1.05 |  |  |  |  |  |  |  |  |
| **11** | 17.37 | 1.10 | 1.10 |  |  |  |  |  |  |  |  |
